# Connected by Boredom: A Systematic Review of the Role of Trait Boredom in Problematic Technology Use

**DOI:** 10.3390/brainsci15080794

**Published:** 2025-07-25

**Authors:** Ginevra Tagliaferri, Manuel Martí-Vilar, Francesca Valeria Frisari, Alessandro Quaglieri, Emanuela Mari, Jessica Burrai, Anna Maria Giannini, Clarissa Cricenti

**Affiliations:** 1 Department of Psychology, Sapienza University of Rome, Via dei Marsi 78, 00185 Rome, Italy; e.mari@uniroma1.it (E.M.); jessica.burrai@uniroma1.it (J.B.); annamaria.giannini@uniroma1.it (A.M.G.); clarissa.cricenti@uniroma1.it (C.C.); 2 Department de Psicologia Bàsica, Faculty of Psychology and Speech Therapy, Universitat de València, Av. Blasco Ibañez, 21, 46010 Valencia, Spain; 3 Department of Psychology of Development and Socialization Processes, Sapienza University of Rome, Via dei Marsi 78, 00185 Rome, Italy; francescavaleria.frisari@uniroma1.it; 4 Department of Human and Social Science, Universitas Mercatorum, Piazza Mattei 10, 00186 Rome, Italy; alessandro.quaglieri@unimercatorum.it

**Keywords:** trait boredom, problematic technology use, technology addictions, social media addiction, internet gaming disorder, internet addiction, gambling, psychological vulnerabilities

## Abstract

**Background/Objectives:** In an increasingly pervasive digital environment, trait boredom has been identified as a key psychological factor in the onset and maintenance of problematic digital technology use. This systematic review aims to investigate the role of trait boredom in digital behavioral addictions, including problematic smartphone use, Internet and social media overuse, and gaming addiction, through theoretical models such as the I-PACE model and the Compensatory Internet Use Theory (CIUT). **Methods:** A systematic literature search was conducted across multiple scientific databases (PsycINFO, Web of Science, PubMed, and Scopus), yielding a total of 4603 records. Following the PRISMA guidelines after duplicate removal and screening based on title and abstract, 152 articles were assessed for full-text eligibility, and 28 studies met the predefined inclusion and exclusion criteria and were included in the final review. **Results:** Findings reveal that trait boredom functions as both a direct and indirect factor in problematic technology use. It serves as a mediator and moderator in the relationship between psychological vulnerabilities (e.g., depression, alexithymia, vulnerable narcissism) and dysfunctional digital behaviors. Furthermore, as an independent variable, it has an influence on technological variables through Fear of Missing Out (FoMO), loneliness, low self-regulation, and dysfunctional metacognitions, while protective factors such as mindfulness and attentional control mitigate its impact. **Conclusions:** Boredom represents a central psychological lever for understanding behavioral addictions in the digital age and should be considered a key target in preventive and therapeutic interventions focused on enhancing self-regulation and meaningful engagement with free time.

## 1. Introduction

Behavioral addictions, also referred to as non-substance or non-chemical addictions, are disorders characterized by an inability to control certain behaviors, resulting in detrimental consequences for an individual’s mental, emotional, physical, or social well-being [1]. Among these are technological addictions, which encompass both passive interactions (e.g., watching television) and active interactions (e.g., computer gaming) between individuals and technological devices [2]. In addition to displaying core features of addiction, such as craving, tolerance, withdrawal, salience, conflict, and relapse, technological addictions are also marked by reinforcing and inductive mechanisms that promote continued engagement with technology [3,4]. In the present article, the terms “problematic technology use,” “technology addiction,” and “behavioral addiction” are used interchangeably, as they all refer to dysfunctional patterns of excessive or uncontrolled engagement with digital devices and environments.

A specific subtype of technological addiction is Internet Addiction, also known as Internet Addiction Disorder (IAD) or problematic Internet use. According to Young [5], IAD can be classified into five behavioral subtypes: compulsive online gambling, cybersexual addiction, information overload, Internet Gaming Disorder (IGD), and addiction to virtual relationships. Over time, additional forms of technological addiction have been identified, such as smartphone addiction and social media addiction (SMA), the latter defined as “the inability to regulate social network use, leading to negative personal and interpersonal outcomes” [6,7]. Among these, IGD-characterized by persistent and recurrent gaming behavior that leads to significant social, occupational, familial, or academic impairment [8]-is the only form officially recognized as a behavioral addiction. It is included in the latest edition of the International Classification of Diseases (ICD-11) under the section on disorders due to addictive behaviors [8] and has been proposed in the DSM-5-TR as a condition for further study [9].

Phenomena such as phubbing and FoMO (Fear of Missing Out) have emerged as contributory factors in the development and maintenance of technological addictions, particularly in the contexts of Internet and social media use [10,11]. These phenomena reflect a constant need for connectivity and a fear of social exclusion, both of which can exacerbate problematic technology use.

Given that these behavioral addictions involve a dysregulation of impulse control, IGD, IAD, and SMA have been found to be associated with various personality traits, including impulsivity, emotional dysregulation, sensation seeking, poor social skills, and social withdrawal [12,13,14], as well as with anxiety and mood-related psychopathologies [10].

In the current digital age, marked by the pervasive presence of technology in nearly every facet of daily life, it is crucial to understand how unlimited access to entertainment, information, and social interaction influences psychological well-being. In this complex landscape, boredom has emerged as a predictive factor for the use and overuse of digital devices and the Internet [15,16].

Boredom, defined as “an aversive state of wanting, but being unable, to engage in satisfying activity” [17] (p. 483), can be conceptualized in two primary forms: state boredom (SB)-a transient, situational experience resulting from insufficient stimulation-and trait boredom (TB)-a chronic personality disposition leading individuals to frequently experience boredom across various contexts [17,18,19]. While state boredom arises from the interaction between person and context [20], trait boredom is considered a stable personality feature influenced by internal psychological factors [21,22].

Although boredom can be temporary and context-dependent, some individuals experience it more frequently, giving rise to the construct of boredom proneness, or trait boredom [21]. Despite their different origins, state and trait boredom are positively correlated, as individuals high in trait boredom are more likely to experience situational boredom [20].

Numerous studies [20,23,24,25,26] indicate that trait boredom has a significant impact on well-being and has been linked to various clinical, psychological, and social problems, such as depression and anxiety, even when controlling for potential confounding variables [27]. Trait boredom is associated with a general sense of emptiness, meaninglessness, and lack of stimulation-features commonly shared with depression. Mercer-Lynn et al. [27] demonstrated that individuals with high levels of trait boredom tend to continuously seek ways to escape boredom but often struggle to find activities that provide purpose or meaning. This cycle of unsuccessful attempts may exacerbate feelings of helplessness and hopelessness, contributing to the development of depression.

Trait boredom can also lead to disengagement from daily activities, fueling cycles of social isolation and emotional withdrawal, two dynamics often implicated in the onset and maintenance of depression. Moreover, trait boredom may function as a disruptive force in maintaining meaningful social relationships, making individuals less likely to engage in rewarding activities or positive interpersonal interactions [12,13,14].

Similarly, trait boredom has been linked to anxiety. Boredom-prone individuals often experience chronic difficulties in sustaining attention, resulting in restlessness. The absence of stimulation or the inability to find meaningful engagement may intensify anxious feelings, as the mind becomes preoccupied with negative thoughts or worries. Anxiety may also be amplified by the sense of wasting time or the awareness of not engaging in productive or fulfilling activities [28].

Chronic boredom may generate a vicious cycle in which individuals attempt to escape the aversive state through maladaptive or risky behaviors, such as substance abuse, compulsive technology use, or other addictive behaviors. These temporary coping strategies ultimately fail to address the underlying problem and may worsen anxiety in the long term.

A key contribution of Mercer-Lynn et al. [27] is the finding that trait boredom can predict psychosocial problems such as depression and anxiety independently of other confounding variables, including personality traits, stressful life events, or preexisting psychological disorders. This suggests that trait boredom is not merely a comorbid symptom, but an active and independent risk factor in the etiology of these conditions.

From a psychological perspective, trait boredom may negatively impact psychosocial well-being through several mechanisms, such as emotional dysregulation (individuals high in trait boredom often struggle to manage their emotions and are more likely to experience frustration, apathy, and dissatisfaction) [29]; lack of meaningful purpose (trait boredom is frequently associated with a lack of meaningful goals or fulfillment in life, which can fuel existential emptiness and anxiety); and low frustration tolerance (boredom-prone individuals typically experience greater discomfort in monotonous or unstimulating situations and have a reduced capacity to tolerate frustration, further contributing to anxiety and depressive symptoms) [27].

### 1.1. Theoretical Models of Boredom

Recent theories and models provide various explanations for the causes and consequences of boredom in relation to the use and overuse of digital devices.

The Meaning-and-Attentional Components (MAC) model [30,31] conceptualizes boredom as arising from the interaction between the attentional demands of a task and the subjective meaning ascribed to it. Individuals may experience boredom when an activity is perceived as meaningless, regardless of its attentional demands (whether too easy or too difficult), or when meaningful activities are perceived as either overly challenging or insufficiently stimulating. Conversely, when meaning and attention are well-balanced, individuals can enter a state of flow, characterized by deep focus and intrinsic enjoyment [30,31].

According to Poels and colleagues [32], boredom may lead to digital media use as a form of escapism or sensation-seeking, or to media multitasking as a way to counteract under-stimulation.

The Boredom Feedback Model (BFM) [33] describes how individuals cope with boredom by engaging in avoidance strategies, such as redirecting attention to external stimuli (e.g., digital media) or through self-reflection. Repeated use of digital media as a means of avoiding boredom may evolve into an anticipatory strategy, whereby even the slightest sense of boredom triggers excessive media use.

This triggering mechanism is closely aligned with the Interaction of Person-Affect-Cognition-Execution (I-PACE) model for addictive behaviors [34] and with the Compensatory Internet Use Theory (CIUT). These models suggest that if individuals repeatedly experience digital media use (e.g., online gaming or smartphone use) as effective in alleviating negative emotions such as boredom, they may increasingly rely on this coping strategy during future episodes of boredom, eventually developing a dependence on these compensatory tools. The I-PACE model [34] emphasizes that in the more advanced stages of addiction, the compensatory use of digital media tends to override its initial rewarding functions, as inhibitory control becomes compromised, further reinforcing the compulsive use of technology in response to boredom. In this context, boredom is regarded as a critical trigger for problematic digital media use, although it is important to note that such tools can themselves become sources of boredom when they fail to provide meaningful or engaging stimulation.

In recent years, several studies [35,36,37] have investigated the role of boredom in the onset of risky behaviors and behavioral addictions, such as substance abuse and problematic use of the Internet or social media [7,38]. To date, however, only one systematic review has examined the relationship between boredom and digital device use [39], and no systematic reviews have focused exclusively on the relationship between trait boredom and the use or misuse of digital devices and the Internet.

Therefore, the present work aims to fill this gap in the literature by exploring how individuals with high levels of boredom proneness may be more vulnerable to the development of technology-related addictions, and how this personality trait may influence psychological and social well-being.

### 1.2. Aim of the Study

The aim of the present systematic review is to synthesize empirical evidence on the relationship between trait boredom and the use of digital media and the Internet. Specifically, the review distinguishes among various forms of technology use, including problematic Internet use/Internet addiction, social media and social networking addiction, online gambling and gaming, problematic smartphone use, and online pornography addiction. Furthermore, the review considers related biopsychosocial characteristics, such as personality traits, mood disorders, sensation seeking, and impulsivity.

Only studies that assessed trait boredom using the Boredom Proneness Scale (BPS) [21], including both the full and short-form versions [40], were included. Studies that measured both state and trait boredom were also retained (e.g., [15,41]). Additionally, boredom was analyzed both as an independent variable and as a mediating factor.

## 2. Materials and Methods

This systematic review was performed according to the recommendations of the “Preferred Reporting Items for Systematic Reviews and Meta-Analyses” (PRISMA) [42,43]. The study was registered in the “International Prospective Register of Systematic Reviews” (PROSPERO) in May 2024 (CRD42024538859), and the detailed protocol is available upon request.

### 2.1. Inclusion Criteria

A systematic review (SR) was carried out after the recommendations of the PRISMA guideline [42] for this type of research. Appendix A includes a checklist following the PRISMA guide (Table A1). A systematic review was conducted by searching four academic databases: PsycINFO (via EBSCOhost), Web of Science, PubMed, and Scopus. The search strategy included keywords related to boredom (“bored*”) and digital media (digital OR internet OR technology OR “social media” OR “social network” OR “smartphone” OR “gaming” OR “shopping online” OR “pornography” OR “cybersexuality” OR “cyber-relationship” OR “information overload” OR “gambling” OR “watching”).

The screening process was carried out using Rayyan. Study selection was performed in two phases: initial screening based on title and abstract, followed by full-text screening by two independent reviewers (GT and CC) according to predefined inclusion and exclusion criteria. Any discrepancies were resolved through discussion until consensus was reached.

Studies were included if they met the following criteria: (1) published in English in peer-reviewed journals; (2) employed a quantitative design (cross-sectional, longitudinal, or cohort); (3) investigated the relationship between trait boredom and technology-related addictions; (4) included participants from the general population; (5) utilized a validated measure of trait boredom.

Studies were excluded if they met the following: (1) were not published in English; (2) were qualitative studies, reviews, meta-analyses, case studies, commentaries, books, book chapters, theses, reports, or conference proceedings; (3) focused on clinical populations; (4) investigated only offline gambling/gaming behaviors; (5) did not use an empirical measure specifically assessing trait boredom (e.g., measures focused on state boredom or combined with other negative affect scales); (6) did not employ an empirical measure for technology-related addictions; (7) did not assess the relationship between trait boredom and technology-related addictions; (8) were conducted in the context of the COVID-19 pandemic. Studies conducted during COVID-19-related lockdowns were excluded to avoid interpretative biases due to the temporary increase in boredom and technology use specifically associated with that historical period [44,45].

The database search yielded a total of 4603 records. After removing duplicates (n = 1770), the titles and abstracts of 2833 records were screened, resulting in the exclusion of 2681 records. Full-text screening was conducted on the remaining 152 articles. Of these, 124 were excluded for the following reasons: non-English language (n = 7), study design not cross-sectional/quantitative (n = 17), no assessment of trait boredom or its relationship with problematic or addictive technology use (n = 92), clinical populations (n = 6), or background articles (n = 2).

**Figure 1:**
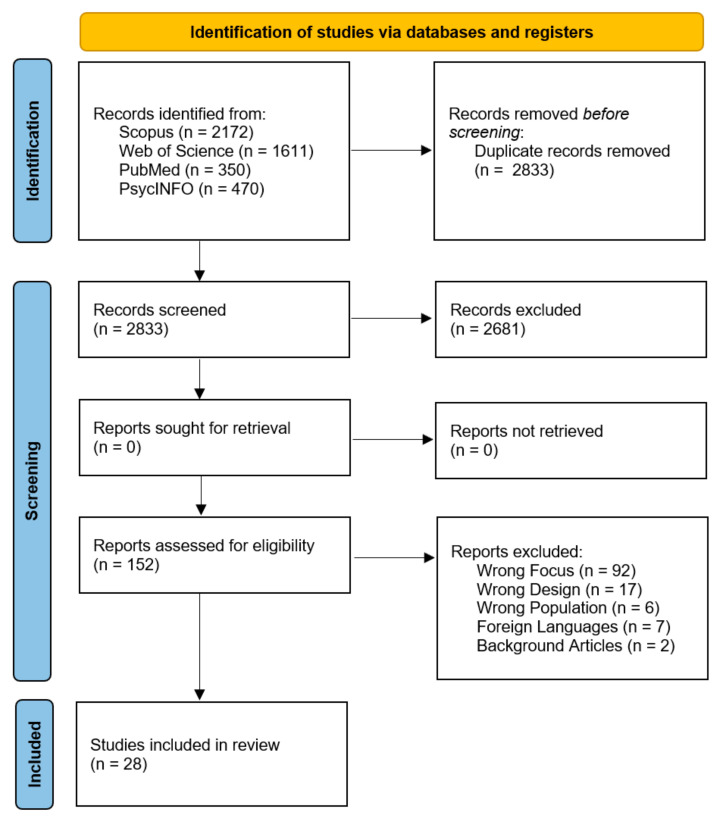
Flowchart of the selection and screening process of the systematic review articles according to the PRISMA criteria.

This screening process resulted in a final inclusion of 28 studies in qualitative synthesis. The PRISMA flow diagram (Figure 1) summarizes the study selection process.

### 2.2. Risk of Bias Assessment

The methodological quality of each study was assessed using the National Institutes of Health (NIH) Quality Assessment Tool for Observational Cohort and Cross-Sectional Studies (https://www.nhlbi.nih.gov/, accessed on 19 September 2024). The same authors who conducted the article selection process independently evaluated the quality of the included studies. Quality ratings were based on the proportion of “yes” responses to the items in the NIH assessment tool.

Specifically, studies were rated as “good” if they received ≥75% positive responses to the NIH tool items (N = 11), as “fair” if they received between 50% and 75% positive responses (N = 15), and as “poor” if they received between 25% and 50% positive responses (N = 2). Studies scoring ≤ 25% were classified as “very poor”; however, no studies met this criterion, and therefore none were excluded based on the quality assessment. In total, 28 studies were included in the qualitative review (see Table 1).

The methodological quality of the studies included was generally acceptable, with 11 studies rated as “good,” 15 as “fair,” and only 2 as “poor.” Most studies clearly stated their research objectives and employed validated instruments for both exposure and outcome measures. However, several common methodological weaknesses were identified. Notably, a majority of studies did not report sample size justifications or conduct power analyses, limiting the statistical robustness of their findings. Furthermore, control for potential confounding variables was often insufficient or absent, particularly in studies rated as “fair” or “poor.” The widespread reliance on cross-sectional designs also limited the capacity to infer temporal or causal relationships. These limitations underscore the need for more rigorous designs and comprehensive reporting in future research. Further information regarding the evaluation of evidence quality and recommendation strength, as conducted using the GRADE methodology, is provided in Table S1.

## 3. Results

### 3.1. Characteristics of the Studies

The 28 studies included in the review were published between 2004 and 2024, with 68% (N = 19) published after 2019. The studies were conducted in Asia (N = 11), America (N = 7), Europe (N = 8), or both in Asia and America (N = 1). Only one study did not specify the country of origin.

The majority of research on this topic has been conducted in Asian contexts, with a notable concentration in China; in fact, all 11 studies in Asia were conducted in China. Overall, 39.3% of the studies included were conducted in Asia, 25.0% in America, and 28.6% in Europe.

In conducting the review, we sought to include all types of study designs; however, the vast majority of studies available in the literature on this topic are cross-sectional. We were able to identify and include only one longitudinal study [65].

The relationship between boredom and various forms of problematic digital media use was investigated across the studies, including problematic Internet use/Internet addiction (N = 5), social media and social networking addiction (N = 6), smartphone addiction (N = 14), gambling addiction (N = 2), and problematic use of online pornography (N = 1).

All studies employed the Boredom Proneness Scale [21] as a measure of trait boredom, with sixteen studies utilizing the short-form version [40].

All the key information extracted from the studies included in the review is summarized in Table 2.

### 3.2. Main Results

In the majority of studies (N = 18), the primary aim was to investigate the direct relationship between trait boredom and technology-related addiction, often in conjunction with other variables such as well-being, social factors, and personality traits. In the remaining studies (N = 10), boredom or addiction was examined as a mediating or moderating variable in the relationship between other factors (e.g., well-being, social dimensions, and personality characteristics).

#### 3.2.1. Boredom and Internet Addiction

The included studies [38,48,54,64] consistently revealed a positive relationship between boredom proneness and problematic or addictive Internet use, regardless of participants’ age. Specifically, Biolcati and colleagues [38] examined differences between adolescents with high (High Boredom, HB) and low boredom proneness (Low Boredom, LB) during their leisure time. The findings indicated that adolescents in the HB group scored higher on the Internet Addiction Test and reported more frequent technology use, whereas those in the LB group engaged more often in offline activities such as sports and reading.

Kiss et al. [14] conducted a study aimed at identifying user profiles based on Internet addiction by examining both risk and protective factors (i.e., boredom proneness, flow state, resilience, self-esteem, self-regulation, and sensation seeking). The results identified four distinct user profiles: strongly protected problematic sensation seekers, not vulnerable balanced users, protected conscious users, and strongly problematic unprotected users. Among these, the first and fourth profiles showed greater vulnerability to problematic Internet use. However, while the strongly problematic unprotected group was characterized by low levels of protective factors, the strongly protected problematic sensation seekers profile exhibited high levels of boredom proneness and sensation seeking.

Problematic or addictive Internet use appears to be associated not only with boredom but also with other psychological variables such as anxiety and depression [54], as well as social and familial loneliness [64], which emerged as stronger predictors than boredom alone. Moreover, Spada et al. [54] observed that when metacognitions were considered, the relationship between boredom and problematic Internet use became non-significant, suggesting that this association may be fully mediated by metacognitive beliefs.

#### 3.2.2. Boredom and Social Network/Social Media Addiction

The included studies revealed a significant positive effect of boredom proneness on general social network addiction [58], problematic Facebook use [15], Internet-communication disorder [51], and Problematic Mobile Social Media Usage [57]. Additionally, several studies identified significant mediating effects of various psychological factors-such as metacognitions [58], desire thinking [58], avoidance of negative feelings through online activities [51], and craving [51,58]-in the relationship between boredom proneness and problematic use of social networks and social media.

Conversely, Bai et al. [55] found that boredom proneness significantly mediated the relationship between mobile social media use and subjective well-being. Furthermore, Pi and colleagues [57] identified distinct profiles of social media users based on levels of Problematic Mobile Social Media Usage. Their results revealed three user groups: no-problem group (26.44%), mild-problem group (56.66%), and severe-problem group (16.91%). Users in the severe-problem group were more likely to report higher levels of Fear of Missing Out (FoMO), a stronger desire for positive online feedback, and higher boredom proneness.

In contrast to these findings, Yao et al. [65] investigated the mediating role of boredom proneness in the relationship between depression, social anxiety, and problematic TikTok use. Their results did not reveal any significant effect of boredom proneness on problematic TikTok use.

#### 3.2.3. Boredom and Gambling Addiction

Regarding gambling addiction, Hopley and Nicki [49] identified boredom proneness-alongside other factors such as dissociation, impulsivity, and negative affective states-as a significant predictor of gambling addiction in a sample of online poker players. Conversely, Mercer and Eastwood [53] found that boredom susceptibility was a significant predictor of gambling disorder, whereas boredom proneness did not show a significant effect. According to Mercer and Eastwood, therefore, boredom is related to problematic gambling when it arises from a state of under-arousal (i.e., boredom susceptibility), rather than from a negative affective state (i.e., boredom proneness).

#### 3.2.4. Boredom and Smartphone Addiction

Several studies have found a direct positive relationship between boredom and problematic smartphone use (PSU) [28,56,60,61,62,63].

In addition, multiple studies reported significant mediating effects of various factors in the relationship between boredom and PSU, including fear of missing out (FoMO) [63], depression [28], smartphone use as a pastime and self-regulation [62], and metacognitions [56]. Specifically, boredom was found to have a positive effect on FoMO [63], depression [28], smartphone use as a pastime [62], and negative metacognitive beliefs regarding uncontrollability and consequences, as well as positive and negative expectancies toward smartphone use. Conversely, boredom had a negative effect on self-regulation [62], which in turn appeared to reduce PSU severity. Except for Wolniewicz et al. [63], who did not report whether a direct effect between boredom and PSU was tested, Casale et al. [56] were the only ones to identify a full mediation effect and to explore the role of cognitive processes.

Additionally, Regan et al. [50] and Yang et al. [28] found a moderating effect of mindfulness and attentional control, respectively, in the relationship between boredom and PSU. Specifically, Regan et al. [50] found a significant positive association between boredom and PSU at low and moderate levels of mindfulness in individuals with low or moderate impulsivity. In contrast, no significant relationship emerged at high levels of impulsiveness. However, for individuals with low mindfulness, an opposite trend was observed, where PSU decreased as boredom proneness increased. Yang et al. [28] found a significant positive association between boredom and PSU only at high levels of attentional control.

Only one study [66] examined the mediating role of PSU in the relationship between boredom, negative emotions, and bedtime procrastination. The results showed significant partial mediation, with positive effects found for all paths investigated.

Most studies focused on the mediating role of boredom in the relationships between psychological symptoms [47,52,62], personality traits and emotional functioning [29,61], psychosocial factors [46,67,128], and PSU.

With regard to personality traits and emotional functioning, Xiao et al. [29] and Ksinan et al. [61] examined the mediating role of boredom in the relationship between alexithymia and narcissism, respectively, and PSU. Xiao et al. [29] found a partial mediation with positive effects across all variables. In contrast, Ksinan et al. [61] found a full mediation effect of boredom in the relationship between vulnerable narcissism (i.e., individuals more likely to avoid social interaction due to lack of positive feedback) and PSU, whereas no significant effects were found for grandiose narcissism (i.e., individuals who seek dominance but depend on others for admiration and reassurance).

Two studies [47,62] examined boredom as a mediator between depression, anxiety, and PSU. Both found a significant mediating effect of boredom in the relationship between depression and PSU, with all effects being positive. However, regarding anxiety, only Elhai et al. [47] reported a significant mediation effect, whereas Wang et al. [62] did not find a significant effect of anxiety on boredom. In both studies, the direct relationship between depression, anxiety, and PSU was not investigated.

Holte et al. [52] reported a significant mediating role of boredom in the relationship between obsessive–compulsive disorder severity and PSU, with all effects being positive.

Regarding psychosocial factors, Elhai et al. [46] and Li et al. [67,128] explored the mediating role of boredom in the relationships between FoMO and loneliness, respectively, and PSU. Elhai et al. [46] identified a partial mediation by negative affect, including boredom, in the relationship between FoMO and PSU, with all effects being positive. Finally, Li et al. [67,128] found that boredom mediated the relationship between loneliness and PSU, with boredom negatively affecting both core self-evaluations (i.e., appraisal of one’s capabilities and self-worth; [128]) and self-control [67].

#### 3.2.5. Boredom and Online Pornography Consumption

Moynihan et al. [59] tested three models in which perceived meaninglessness was treated as the independent variable, and variables related to problematic pornography use (i.e., frequency of pornography use, excitement seeking, sexual pleasure) were considered as dependent variables. Emotional avoidance and boredom proneness were included as mediators. In each model, the direct effect of perceived meaninglessness on the dependent variables was not significant, whereas the indirect effect through the mediators was significant. Specifically, boredom proneness had a significant positive effect on emotional avoidance.

## 4. Discussion

The present systematic review aimed to examine the relationship between trait boredom and the use of digital media and the Internet, distinguishing between different technological devices (problematic use/addiction to the Internet, addiction to social media and social networks, gambling and gaming, problematic smartphone use, and online pornography addiction) and biopsychosocial characteristics (e.g., personality traits, mood disorders, sensation seeking, impulsivity). In the current digital era, marked by the pervasive presence of technology in everyday life, boredom has emerged as a significant predictor for the use and overuse of digital devices and the Internet [15,16].

### 4.1. Boredom and Internet Addiction

The findings emerging from the analyzed studies confirm the significant role of boredom, particularly trait boredom, as a significant predictor for problematic Internet use, in line with previous literature (e.g., [15,16,27]). The fact that this association is observed regardless of age suggests that boredom is a cross-cutting construct relevant to the development of technological addictions.

Biolcati et al. [38] highlight how adolescents with high trait boredom tend to rely more heavily on Internet use during their free time, whereas those with low boredom proneness prefer more stimulating and socially shared activities (e.g., sports, reading). This evidence aligns with theoretical frameworks, particularly the MAC model [30,31], which posits that boredom arises from a mismatch between perceived meaning and attentional demand. Adolescents in the high boredom group may perceive offline activities as insufficiently engaging or meaningful, thus turning to digital media as an apparent relief from this cognitive–emotional dissonance.

Kiss et al. [14], on the other hand, identify user profiles based on a combination of risk and protective factors. Notably, the “strongly protected problematic sensation seekers” profile demonstrates that even individuals with strong psychological resources may be vulnerable to dysfunctional Internet use when high boredom proneness and sensation-seeking tendencies are present. This finding is consistent with the I-PACE model [34], which suggests that dispositional traits (such as trait boredom) interact with affective and cognitive processes, contributing to the gradual loss of control over digital media use. A critical issue highlighted by Spada et al. [54] is that the relationship between boredom and problematic Internet use loses statistical significance when dysfunctional metacognitions are considered-that is, beliefs about the Internet as a tool for regulating negative emotions or thoughts. This suggests that boredom may indirectly promote dysfunctional behaviors through the mediation of metacognitive coping strategies.

These findings are consistent with the Compensatory Internet Use Theory (CIUT), which posits that the Internet is used to compensate for negative internal states such as boredom, anxiety, and loneliness. Furthermore, as noted by Nichols & Nicki [64], relational factors such as family and social loneliness appear to be stronger predictors with problematic Internet use rather than boredom alone. This underscores that trait boredom may be part of a broader framework involving additional psychosocial and relational factors, in line with the view that it is associated with difficulties in emotional regulation, social withdrawal, and impaired interpersonal functioning [12,13]. Overall, the results suggest that trait boredom might take effect as a vulnerability factor, particularly when combined with other risk elements (e.g., low self-regulation, low resilience, low self-esteem, high sensation seeking), thereby contributing to the development of problematic Internet-related behaviors. However, its influence appears to be partially mediated or moderated by cognitive (e.g., metacognitive beliefs) and relational factors (e.g., loneliness), highlighting the need for a multifactorial approach in the prevention and treatment of technology-related addictions.

### 4.2. Boredom and Social Network/Social Media Addiction

The data confirm a significant and positive relationship between boredom proneness and problematic social media use, a result consistent with the theoretical corpus on the role of boredom in dysfunctional online behaviors [15,51,57,58]. These findings are situated within the broader theoretical framework of the I-PACE model [34], which emphasizes how personality traits, including trait boredom, may function as significant predictors in the cycle leading to problematic technology use. This association has been observed across various domains: from general social network addiction [58] and problematic Facebook use [15], to more specific manifestations such as online communication disorder [51] and Problematic Mobile Social Media Usage [57]. This suggests that boredom proneness is not limited to influencing a single type of dysfunctional digital behavior but may be related a diverse problematic pattern related to the social media sphere. A particularly relevant aspect concerns the mediating role of cognitive and motivational processes in the relationship between boredom and problematic use. Specifically, metacognition and desire thinking [58], as well as craving and emotional avoidance [51], have proven to be significant mediators. This implies that it is not boredom per se that leads to problematic use, but rather the way in which individuals interpret and regulate their experience of boredom.

This result is supported by theories suggesting that boredom exerts an indirect effect: it is not the mere experience of emptiness or lack of stimulation that triggers problematic behavior, but the attempt to cope with it through dysfunctional cognitive strategies (e.g., repetitive thinking, uncontrollable desire, emotional avoidance).

The Compensatory Internet Use Theory (CIUT) is especially relevant here, as it proposes that social media use can serve as a coping strategy to compensate for unpleasant internal states such as boredom or dissatisfaction. Bai et al. [55], moreover, highlight a mediating effect of boredom between social media use and subjective well-being, suggesting that boredom not only drives excessive use but can also mediate the psychological consequences of such use. Additionally, research by Pi et al. [57] offers valuable insight into individual differences in problematic use. The identification of three user profiles-from “non-problematic” to “severely problematic”-underscores how high levels of FoMO, desire for positive feedback, and boredom proneness are key markers of the problematic profile. This supports the idea that boredom does not act in isolation but interweaves with other motivational and affective factors, reinforcing the tendency toward excessive and dysfunctional social media use. However, findings from Yao et al. [65] introduce an element of complexity. In their study, boredom proneness did not play a significant role in the relationship between depression, social anxiety, and problematic TikTok use. This may be explained by various factors: the nature of TikTok content and its use experience-highly oriented toward immediate rewards and sensory gratification-may fulfill motivations different from those underlying more text- or relationship-based social media. It is possible that, in this context, social anxiety and depression act as dominant factors, overshadowing the contribution of boredom in explaining dysfunctional use. Finally, this may reflect methodological differences (e.g., measurement tools, sample characteristics, cultural context) or suggest that boredom is not universally correlated, but that its influence varies depending on the platform and the user’s psychological profile.

### 4.3. Boredom and Gambling Addiction

In the context of gambling addiction, the relationship between boredom and problematic behaviors appears less linear than what has been observed for problematic Internet or social media use. Although limited, the available studies offer interesting and complementary perspectives on the role of boredom as a strongly correlated factor in gambling addiction, suggesting that not all forms of boredom contribute equally to dysfunctional behavior.

The study by Hopley and Nicki [49] found that boredom proneness, alongside variables such as dissociation, impulsivity, and negative affective states, was correlated with gambling addiction in a sample of online poker players. This finding aligns with a dispositional perspective on boredom: individuals who frequently experience boredom in daily life may be more inclined to seek intense stimulation to fill an experiential void, and gambling, with its high potential for excitement and intermittent reward, can serve as an effective means of self-stimulation.

However, the results of Mercer and Eastwood [53] introduce an important conceptual distinction between two often conflated constructs: boredom proneness, understood as a stable tendency to experience boredom, often associated with negative affective states such as frustration or dissatisfaction, and boredom susceptibility, which instead reflects a hypersensitivity to under-stimulating environments, linked to low arousal states and a desire for novelty.

In their study, Mercer and Eastwood [53] found that only boredom susceptibility was associated of gambling disorder. This suggests that, in the case of gambling, it is the seeking stimulation, rather than the regulation of negative emotions, that primarily motivates problematic behavior. It is worth highlighting that on one hand, boredom proneness may drive individuals toward compensatory activities aimed at alleviating emotional distress, as is often observed in problematic Internet and social media use. On the other hand, boredom susceptibility implies a profile more closely aligned with sensation seeking or cognitive impulsivity, traits that are significant predictors of gambling addiction, especially in its faster and more stimulating forms (e.g., slot machines, online poker). These findings suggest that the motivational function of boredom may vary depending on the behavioral context: in social media and Internet use, boredom may act as an emotional trigger associated with avoidant coping or rumination; in contrast, it appears to function more as a motivational driver, prompting the search for activation and immediate gratification.

### 4.4. Boredom and Smartphone Addiction

The results examined confirm and expand the theoretical hypotheses regarding the central role of boredom, particularly boredom as a character trait, associated with individuals with problematic smartphone use (PSU). The collected evidence consistently shows a direct positive relationship between boredom and PSU [28,56,60,61,62,63], in line with the I-PACE model [34] and the Compensatory Internet Use Theory [145]. According to these frameworks, boredom-especially when chronic-acts as a negative emotional trigger that drives individuals to use digital devices as a means of compensating for unpleasant internal states. The MAC Model [30,31] posits that boredom arises when the ongoing activity is perceived as lacking in meaning or insufficiently engaging relative to the individual’s attentional capacity. In this regard, the smartphone serves as a cognitively accessible “shortcut,” offering varied and immediate stimuli without requiring real engagement or purpose-yet reinforcing dysfunctional usage cycles. This is reflected in findings that show smartphone use as a pastime [62] acting as a compensatory mechanism mediated by boredom. Notably, several psychological variables have been identified as significant mediators in the relationship between boredom and PSU-namely FoMO [63], depression [28], and instrumental smartphone use as a pastime and poor self-regulation [62], as well as dysfunctional metacognitions [56].

These results highlight that boredom does not operate solely as a direct factor but fuels secondary processes that intensify smartphone use: on one hand, by amplifying negative emotional states (e.g., depression, FoMO), and on the other, by undermining self-regulatory abilities and critical reflection on behavior [56].

Interestingly, Casale et al. [56] were the only researchers to observe a full mediation effect and to include metacognitive processes, emphasizing the need to explore implicit beliefs regarding smartphone use, alongside affective states. Two studies [28,50] examined moderating factors in the boredom–PSU relationship. Specifically, mindfulness and attentional control emerged as significant moderators: on one hand, high levels of attentional control appear to increase sensitivity to boredom, possibly because they enhance awareness of internal states without necessarily improving regulation capacities [28]; on the other hand, mindfulness appears to buffer the relationship between boredom and PSU, suggesting that greater present-moment awareness may mitigate impulsive compensatory smartphone use [50].

Other studies highlight the mediating role of boredom in the relationship between dispositional traits (e.g., narcissism, alexithymia), emotional functioning, and PSU [29,61]. Specifically, boredom fully mediates the link between vulnerable narcissism and PSU [61], indicating that individuals with a fragile sense of self may use smartphones to escape social under-stimulation or a lack of external validation. In the case of alexithymia and PSU, the partial mediation of boredom [29] suggests that difficulty identifying and regulating emotions may foster chronic dissatisfaction and avoidance behaviors such as excessive smartphone use. Regarding the mediating effects of boredom in the relationship between depression, anxiety, OCD, and PSU [47,52,60], these findings support the hypothesis that boredom is not merely a co-occurring symptom but an active factor [27] that facilitates the impairment of psychosocial functioning. The observation that this mechanism also applies to individuals with obsessive–compulsive disorder [52] reinforces the importance of considering boredom as a clinically relevant variable, rather than merely a secondary outcome. Finally, boredom’s mediating role in the relationship between loneliness, core self-evaluations, self-control [67,128], and PSU underscores how boredom undermines self-regulatory mechanisms, contributing to a negative self-view and reduced self-control capacity. This is fully consistent with the cyclical dynamics described in the CIUT and I-PACE models, in which chronic boredom is identified as a significant predictor of dysfunctional behavior.

### 4.5. Boredom and Online Pornography Consumption

The study by Moynihan et al. [59] illustrates how perceived meaninglessness and trait boredom are correlated with the use of pornography as an emotional avoidance strategy. This finding aligns with the I-PACE and Compensatory Internet Use Theory (CIUT) frameworks [34], which posit that digital media, including pornography, are often used to alleviate negative emotional states such as boredom. Boredom, conceptualized both as a stable trait and a transient state, could drives individuals to seek external stimulation to escape emotional emptiness, supporting the Boredom Feedback Model (BMF) [33]. Moynihan et al. [59] highlights that trait boredom is particularly associated with compensatory behaviors such as problematic pornography use and is further linked to psychosocial difficulties, including depression and anxiety. This mechanism of “compensatory boredom” entails risks of addiction, consistent with models that conceptualize boredom as a key factor for dysfunctional media-related behaviors.

## 5. Conclusions

The findings analyzed highlight boredom as a key construct in the context of digital behavioral addictions, functioning both as a direct predisposing factor and as a mediator between psychological vulnerabilities and dysfunctional behaviors. Although the specific mechanisms vary across domains (e.g., problematic use of the Internet, social media, smartphones, gambling, or pornography), boredom consistently emerges as a negative emotional state that triggers the pursuit of immediate gratification and affective regulation through digital means [46,54,59]. Its influence is particularly evident in mediation models, where it serves as an explanatory link between personality traits (e.g., vulnerable narcissism, alexithymia) [29,61], psychopathological symptoms (e.g., depression, loneliness) [47,60,67,128], and problematic use, suggesting a bridging function between internal distress and maladaptive coping strategies. Moreover, boredom shows a positive association with variables such as FoMO [63], emotional avoidance [51,59], dysfunctional metacognitions [56,58], and the use of smartphones as a pastime [62], outlining a cognitively and emotionally vulnerable profile. However, the presence of individual resources such as mindfulness or attentional control [28,50] appears to moderate this relationship, attenuating the impact of boredom on problematic behaviors. These findings support the hypothesis that boredom should not be considered merely a behavioral antecedent but rather a multidimensional psychological construct, whose impact depends on its interaction with motivational, regulatory, and cognitive factors. Considering this, it is essential for prevention and intervention programs to not only target a reduction in dysfunctional technology use but also to promote boredom tolerance, emotional regulation, and the development of personal meaning, especially among clinically or socially vulnerable populations [52,59]. Additional graphical representations illustrating the mediating and moderating role of trait boredom are provided in Supplementary Material S2.

Despite the richness of the available data, several recurring limitations must be acknowledged in the reviewed studies. First, the predominant use of cross-sectional research designs prevents definitive conclusions regarding the directionality and causality of the observed relationships. This methodological constraint should be further emphasized, as it significantly limits the ability to infer dynamic or developmental processes over time.

Second, the reliance on self-report measures exposes findings to biases related to social desirability and memory recall, thereby compromising internal validity. Third, the frequent use of non-representative samples, often composed of university students, restricts the generalizability of the findings to more diverse populations. Future research should aim to include broader demographic groups, varying in age, socioeconomic background, and educational level.

Fourth, although this review includes studies conducted in different countries, little attention has been paid to cross-cultural differences in how boredom is experienced and how it relates to technology use. Cultural norms and values may moderate these relationships, and their neglect limits the ecological validity of the conclusions. Finally, the lack of a systematic consideration of contextual factors further impedes a comprehensive understanding of how boredom interacts with digital behaviors across different social environments.

Considering these limitations, several future research directions emerge. Longitudinal designs are recommended to examine the developmental trajectories between boredom and problematic behaviors, as well as the adoption of multimodal methodological approaches (e.g., physiological measures) to overcome the constraints of self-reported data. Another priority involves diversifying samples to assess cultural and demographic differences. Additionally, a meta-analytic approach represents a valuable future direction to quantitatively examine the relationship between boredom and problematic use of digital devices. Such an analysis could offer deeper insights into the strength and variability of this association across different contexts. However, before conducting a meta-analysis, it will be essential to carefully identify and define potential moderators, such as age, gender, geographic region, type of digital addiction, and study quality, in order to ensure a robust and meaningful synthesis of the available evidence.

Future efforts should focus on the development and empirical validation of targeted interventions that address boredom and enhance self-regulatory capacities, with the goal of preventing or mitigating the onset of behavioral addictions. At present, cognitive behavioral therapy (CBT) remains the primary therapeutic approach for conditions such as Internet Gaming Disorder (IGD) and Internet addiction. CBT often incorporates emotion-focused components, including techniques drawn from mindfulness-based interventions [146]. Additional treatment modalities include family therapy [147], particularly indicated for adolescents and young adults, and pharmacological interventions [148], which may be selectively employed in cases involving psychiatric comorbidities such as attention deficit/hyperactivity disorder (ADHD) and major depressive disorder [149,150].

It is important to note that, even in the absence of clinically defined pathology, engagement with social networking platforms may elicit stress and negative affective states driven by social pressures and fear of negative evaluation. In response, individuals often adopt coping mechanisms that-while initially adaptive-can, in cases of excessive or dysregulated use, evolve into maladaptive patterns characteristic of behavioral addiction [151,152,153,154,155]. In these cases, emotion-focused coping strategies, especially those centered on avoidance, are frequently observed.

Within this framework, there is a growing need to expand traditional therapeutic paradigms by incorporating interventions that promote adaptive coping skills and foster emotional awareness and regulation. Targeting emotions commonly associated with problematic technology use, such as boredom, may serve as a critical clinical leverage point, potentially enhancing both the efficacy and long-term sustainability of treatment outcomes [151,153,156].

Finally, further research should investigate the mediating and moderating mechanisms through which boredom exerts its effects, exploring the role of psychological variables such as FoMO, depression, self-regulation, and metacognitions [28,56,62,63].

## Figures and Tables

**Table 1:** Results of quality assessment of the studies.

Authors	Q 1.	Q 2.	Q 3.	Q 4.	Q 5.	Q 6.	Q 7.	Q 8.	Q 9.	Q 10.	Q 11.	Q 12.	Q 13.	Q 14.	Quality Rating
[46]	Y	Y	Y	N	Y	N	N	Y	Y	NA	Y	Y	NA	Y	Good
[47]	Y	Y	Y	N	Y	N	N	Y	Y	NA	Y	Y	NA	Y	Good
[38]	Y	Y	Y	N	Y	N	N	Y	Y	NA	Y	N	NA	N	Fair
[48]	Y	Y	Y	N	Y	N	N	Y	Y	NA	Y	Y	NA	N	Fair
[49]	Y	Y	Y	N	Y	N	N	Y	Y	NA	Y	Y	NA	N	Fair
[50]	Y	Y	Y	Y	Y	N	N	Y	Y	NA	Y	Y	NA	Y	Good
[51]	Y	Y	Y	N	Y	N	N	Y	Y	NA	Y	NR	NA	Y	Fair
[52]	Y	Y	Y	N	Y	N	N	Y	Y	NA	Y	Y	NA	Y	Good
[53]	Y	Y	Y	N	Y	N	N	Y	Y	NA	Y	Y	NA	N	Fair
[54]	Y	Y	Y	Y	Y	N	N	Y	Y	NA	Y	NR	NA	N	Fair
[55]	Y	N	Y	N	Y	N	N	Y	Y	NA	Y	NR	NA	N	Fair
[56]	Y	Y	NR	Y	Y	N	N	Y	Y	NA	Y	Y	NA	Y	Good
[57]	Y	Y	Y	Y	Y	N	N	Y	Y	NA	Y	Y	NA	N	Good
[58]	Y	Y	Y	N	Y	N	N	Y	Y	NA	Y	Y	NA	Y	Good
[59]	Y	Y	Y	N	Y	N	N	Y	Y	NA	Y	NR	NA	N	Fair
[15]	Y	Y	NR	N	Y	N	N	Y	N	NA	Y	NR	NA	Y	Poor
[60]	Y	Y	Y	N	Y	N	N	Y	Y	NA	Y	Y	NA	Y	Good
[61]	Y	Y	NR	N	Y	N	N	Y	Y	NA	Y	Y	NA	Y	Fair
[62]	Y	Y	NR	N	Y	N	N	Y	N	NA	N	Y	NA	N	Fair
[63]	Y	Y	Y	N	Y	N	N	Y	Y	NA	Y	Y	NA	Y	Good
[14]	Y	Y	Y	N	N	N	N	Y	Y	NA	Y	Y	NA	N	Fair
[64]	Y	Y	Y	N	N	N	N	Y	Y	NA	Y	NR	NA	Y	Fair
[65]	Y	Y	Y	Y	Y	Y	Y	Y	Y	N	Y	Y	Y	Y	Good
[66]	Y	Y	NR	N	Y	N	N	Y	Y	NA	Y	NR	NA	Y	Poor
[67]	Y	Y	Y	N	Y	N	N	Y	Y	NA	Y	NR	NA	N	Fair
[26]	Y	Y	Y	N	Y	N	N	Y	Y	NA	Y	NR	NA	N	Fair
[29]	Y	Y	Y	N	Y	N	N	Y	Y	NA	Y	Y	NA	Y	Good
[28]	Y	Y	NR	N	Y	N	N	Y	Y	NA	Y	N	NA	Y	Fair

Note. The quality of included studies was assessed using the National Institutes of Health (NIH) Quality Assessment tool for Observational Cohort and Cross-Sectional Studies “https://www.nhlbi.nih.gov/health-topics/study-quality-assessment-tools” (accessed on 19 September 2024). Q 1. Was the research question or objective in this paper clearly stated? Q 2. Was the study population clearly specified and defined? Q 3. Was the participation rate of eligible persons at least 50%? Q 4. Were all the subjects selected or recruited from the same or similar populations (including the same time period)? Were the inclusion and exclusion criteria for being in the study prespecified and applied uniformly to all participants? Q 5. Was a sample size justification, power description, or variance and effect estimates provided? Q 6. For the analyses in this paper, were the exposure(s) of interest measured prior to the outcome(s) being measured? Q 7. Was the timeframe sufficient so that one could reasonably expect to see an association between exposure and outcome if it existed? Q 8. For exposures that can vary in amount or level, did the study examine different levels of the exposure as related to the outcome (e.g., categories of exposure, or exposure measured as a continuous variable)? Q 9. Were the exposure measures (independent variables) clearly defined, valid, reliable, and implemented consistently across all study participants? Q 10. Was the exposure(s) assessed more than once over time? Q 11. Were the outcome measures (dependent variables) clearly defined, valid, reliable, and implemented consistently across all study participants? Q 12. Were the outcome assessors blinded to the exposure status of participants? Q 13. Was loss to follow-up after baseline 20% or less? Q 14. Were key potential confounding variables measured and adjusted statistically for their impact on the relationship between exposure(s) and outcome(s)? Q: question; NA: not applicable; NR: not reported; N: no; Y: yes.

**Table 2:** Information of the articles selected for the review.

Author Country	Sample Size (n)	Mean Age (SD)	Percentage of Female (%)	Study Design	Addiction Investigated	Tools for Measuring Boredom	Other Tools
[64] Canada	207	21 (1.73)	42.5%	Cross- sectional study	Internet Addiction	Boredom Proneness Scale (BPS) [21]	▪ Internet Addiction Scale (IAS) [64] ▪ Social and Emotional Loneliness Scale (SELSA) [68]
[54] UK	97	23.3 (3.0)	41%	Cross- sectional study	Problematic Internet Use (PIU)	Boredom Proneness Scale (BPS) [21]	▪ Hospital Anxiety and Depression Scale (HADS) [69] ▪ Metacognitions Questionnaire 30 (MCQ-30) [70] ▪ The Internet Addiction Test (IAT) [5]
[53] Canada	202	22.5 (5.9)	68%	Cross- sectional study	Problematic Gambling Behavior	Boredom Proneness Scale (BPS) [21] Boredom Susceptibility Scale (ZBS) [71]	▪ The Sensitivity to Punishment and Sensitivity to Reward Questionnaire (SPSRQ) [72] ▪ The Canadian Problem Gambling Index (CPGI) [73]
[49] Canada	179	30 (10.25)	3.91%	Cross- sectional study	Gambling/Online Poker Playing	Boredom Proneness Scale-short form (BPS-SF) [40]	▪ Five-item Dissociation Questionnaire (DQ) [74] ▪ Barratt Impulsivity Scale–Short Form (BIS-SF) [75] ▪ Depression Anxiety Stress Scale-21 (DASS-21) [76] ▪ Problem Gambling Severity Index (PGSI) [73]
[48] USA	164	26.86 (7.88)	73.8%	Cross- sectional study	Problematic Internet Use (PIU)	Boredom Proneness Scale (BPS) [21]	▪ Problem Internet Use Questionnaire (PIUQ) [77] ▪ The UCLA Loneliness Scale Version 3 [78] ▪ The Distress Tolerance Scale (DTS) [79] ▪ Academic Performance
[38] Italy	478	16.31 (1.47)	40%	Cross- sectional study	Internet Addiction	Boredom Proneness Scale (BPS) [21]	▪ Frequency of free time activities; substance use ▪ Positive Drinking Expectancy Scale (PDMS) [80] ▪ The Internet Addiction Test (IAT) [5]
[51] Germany	148	25.61 (8.94)	61%	Cross- sectional study	Internet Communication Disorder	Boredom Proneness Scale (BPS) [21]	▪ Short Internet Addiction Test for Internet-Communication Disorder (s-IAT-ICD) [81] ▪ Cue-reactivity paradigm with online-communication-related images [82] ▪ Desire of Alcohol Questionnaire-modified for smartphone use [83] ▪ Internet-Use Expectancies Scale-modified for online communication (IUES) [82,84]
[47] China	298	19.45 (2.17)	76.8%	Cross- sectional study	Problematic Smartphone Use (PSU)	Boredom Proneness Scale-short form (BPS-SF) [40]	▪ Smartphone use frequency (SUF) [85] ▪ Smartphone Addiction Scale (SAS) [86] ▪ Fear of missing out Scale (FoMOs) [87] ▪ Depression Anxiety Stress Scale-21 (DASS-21) [76]
[46] China, Saudi Arabia and USA	296	19.44 (2.16)	76.7%	Cross- sectional study	Smartphone Use and Problematic Smartphone Use (PSU)	Boredom Proneness Scale-short form (BPS-SF) [40]	▪ Smartphone use frequency scale [85] ▪ Process and social use scale [88] ▪ Smartphone Addiction Scale (SAS) [86] ▪ Fear of missing out scale (FoMOs) [87] ▪ Depression Anxiety Stress Scale -21 (DASS-21) [76] ▪ Ruminative thought style questionnaire (RTSQ) [89]
[61] --	532	23.33 (1.75)	54.9%	Cross- sectional study	Compulsive Smartphone Use	Boredom Proneness Scale (BPS) [21]	▪ Narcissism Personality Inventory scale (NPI-16) [90] ▪ Hypersensitive Narcissism Scale (HSNS) [91] ▪ Compulsive usage of smartphones scale [92]
[14] Hungary	249	22.5 (3.5)	62.2%	Cross- sectional study	Problematic Internet and Smartphone Use	Boredom Proneness Scale (BPS) [21]	▪ Smartphone Addiction Inventory (SPAI) [93] Hungarian version: [94] ▪ Problematic Internet Use Questionnaire (PIU-Q) [77] ▪ Brief Sensation Seeking Scale (BSSS-8) [95] Hungarian version: [96] ▪ Rosenberg Self-Esteem Scale (RSES) [97] ▪ Self-Regulation Scale [98] ▪ Flow State Questionnaire [99] ▪ 10-item Connor–Davidson Resilience Scale (CD-RISC-10) [100,101] Hungarian version: [102]
[62] China	442	--	54.5%	Cross- sectional study	Smartphone Addiction	Boredom Proneness Scale (BPS) [21]	▪ Sensation seeking scale (SSS) [103] ▪ Pastime scale [104] ▪ Flow experience [105] ▪ Self-regulation scale [106] ▪ The 10-item Smartphone Addiction Scale-Short Version (SAS-SV) [86]
[28] China	1099	20.04 (1.25)	59.6%	Cross- sectional study	Problematic Mobile Phone Use (PMPU)	Boredom Proneness Scale-short form (BPS-SF) [40]	▪ Center for Epidemiologic Studies Depression Scale (CES-D) [107] ▪ The Mobile Phone Addiction Index (MPAI) [108] ▪ The Attentional Control Scale [109]
[63] USA	297	19.70 (3.96)	72.1%	Cross- sectional study	Problematic Smartphone Use (PSU)	Boredom Proneness Scale-short form (BPS-SF) [40]	▪ Fear of missing out scale (FoMOs) [87] ▪ Smartphone addiction scale [86] ▪ Smartphone use frequency scale [85] ▪ Depression Anxiety Stress Scale-21 (DASS-21) [76]
[60] China	1097	19.38 (1.18)	81.9%	Cross- sectional study	Problematic Smartphone Use (PSU)	Boredom Proneness Scale (BPS) [21]	▪ Smartphone Addiction Scale-Short Version (SAS-SV) [86] ▪ Smartphone Use Frequency Scale (SUF) [85] ▪ Depression Anxiety Stress Scale-21 (DASS-21) [76] ▪ Ruminative Responses Scale (RRS) [110]
[50] USA	135	19.15 (1.22)	68.1%	Cross- sectional study	Problematic Smartphone Use (PSU)	Boredom Proneness Scale (BPS) [21]	▪ The Short Impulsive Behavior Scale (UPPS-P) [111] ▪ The Anxiety/Dependence on Technology subscale of the Media and Technology Usage and Attitudes Scale (MTUAS) [112] ▪ The Mindful Attention Awareness Scale (MAAS) [113] ▪ Self-report allowed for participants to document time spent on each of their iPhone applications in the prior week ▪ The Smartphone Addiction Inventory (SPAI) [93]
[26] China	1078	20 (1.10)	28%	Cross- sectional study	Mobile Phone Addiction	Boredom Proneness Scale-short form (BPS-SF) [40]	▪ Loneliness Self-reporting Scale (UCLA) [78] ▪ Self-control Scale (SCS) [114] ▪ Mobile Phone Addiction Index (MPAI) [108]
[29] China	1267	20.36 (0.97)	59.19%	Cross- sectional study	Problematic Mobile Phone Use (PMPU)	Boredom Proneness Scale-short form (BPS-SF) [40]	▪ Toronto Alexithymia-20 Scale (TAS-20) [115] ▪ Mobile Phone Addiction Index (MPAI) [108] ▪ Social Interaction Anxiousness Scale (SIAS) [116]
[55] China	656	22.13 (4.36)	63.9%	Cross- sectional study	Social Media Use	Boredom Proneness Scale (BPS) [21]	▪ The Problematic Mobile Social Media Usage Assessment Questionnaire [55] ▪ The Overall Happiness Scale [117]
[56] Italy and UK	535	27.38 (9.05)	71.2%	Cross- sectional study	Problematic Smartphone Use (PSU)	Boredom Proneness Scale-short form (BPS-SF) [40]	▪ Depression Anxiety Stress Scales-21 (DASS-21) Italian version [76,118] ▪ Barratt Impulsiveness Scale-15 (BIS-15) Italian version [75,119] ▪ Metacognitions about Smartphone Use Questionnaire (MSUQ) [120] ▪ Smartphone Use Expectancies Scale (SUES) [10] ▪ Smartphone Addiction Scale-Short Version (SAS-SV) Italian version [86,121]
[15] Italy	169	17.13 (1.61)	43%	Cross- sectional study	Problematic Use of Facebook (PFU)	Boredom Proneness Scale-short form (BPS-SF) [40]	▪ The Bergen Facebook Addiction Scale (BFAS) Italian version [122,123] ▪ The Multidimensional State Boredom Scale-Short Form (MSBS-SF) [124]
[67] China	1267	20.40 (0.97)	67.3%	Cross- sectional study	Smartphone Addiction	Boredom Proneness Scale-short form (BPS-SF) [40]	▪ Core self-evaluations scale (CSES) [125] ▪ Mobile phone addiction scale (MPAI-17) [108] ▪ Loneliness Self-reporting Scale (UCLA-20) [78]
[59] UK	179	30.22 (10.70)	41.9%	Cross- sectional study	Pornography Use	Boredom Proneness Scale-short form (BPS-SF) [40]	▪ Meaning in Life Questionnaire-Presence of Meaning Subscale [126] ▪ Pornography Consumption Inventory (PCI)-Subscales: Emotional Avoidance, Excitement Seeking, and Sexual Pleasure [127]
[66] China	668	20.36 (1.69)	64.97%	Cross- sectional study	Mobile Phone Addiction	Boredom Proneness Scale- short form (BPS-SF; Chinese version: [128])	▪ Bedtime procrastination scale (BPS; Chinese version: [129]) ▪ Mobile phone addiction tendency scale (MPATS; Chinese version: [130]) ▪ Depression Anxiety Stress Scale-21 (DASS-21; Chinese version: [131])
[65] China	T1: 822 T2: 715	27.5 (5.93)	65.3%	Longitudinal study	Problematic TikTok Use	Boredom Proneness Scale-short form (BPS-SF) [40]	▪ The Patient Health Questionnaire-9 (PHQ-9; [132] Chinese version: [133]) ▪ The Social Interaction Anxiety Scale (SIAS; [134] Chinese version: [135]) ▪ The Distress Intolerance Scale (DIS) [136] ▪ Daily TikTok use time ▪ The Smartphone Addiction Scale-Short Version (SAS-SV; [86] Chinese version: [104])
[58] Italy	529	32.46 (13.34)	62.9%	Cross- sectional study	Problematic Social Networking Sites Use (PSNSU)	Boredom Proneness Scale-short form (BPS-SF) [40]	▪ The Perth Emotional Reactivity Scale-Short Form (PERS) [137] ▪ Fear of Missing Out Scale (FoMOs; [87] Italian version: [120]) ▪ Metacognitions about Desire Thinking Questionnaire (MDTQ) [138] ▪ Desire Thinking Questionnaire (DTQ) [139] ▪ Penn Alcohol Craving Scale (PACS-SNSs) [140,141] ▪ Bergen Social Media Addiction Scale (BSMAS) [122]
[57] China	2591	20.21 (1.53)	57.5%	Cross- sectional study	Problematic Mobile Social Media Use (PMSMU)	Boredom Proneness Scale-short form (BPS-SF) [40]	▪ Problematic Mobile Social Media Use Scale (PMSMUS) [55] ▪ Fear of Missing Out scale (FoMOs) [87,128] ▪ The Online Positive Feedback Scale [142]
[52] USA	424	38.44 (11.46)	44.3%	Cross- sectional study	Problematic Smartphone Use (PSU)	Boredom Proneness Scale-short form (BPS-SF) [40]	▪ Yale–Brown obsessive–compulsive scale (Y-BOCS) [143] ▪ Fear of missing out Scale (FoMOs) [87] ▪ Intolerance for uncertainty scale-short version-inhibitory anxiety Subscale [144] ▪ Smartphone addiction scale-short version (SAS-SV) [86]

## Data Availability

The data presented in this study are available on request from the corresponding author.

## Supplementary Materials

The following supporting information can be downloaded at: https://www.mdpi.com/article/10.3390/brainsci15080794/s1, Table S1: GRADE System; Figure S1: Graphical representation illustrating the mediating and moderating role of trait boredom.

## Abbreviations

The following abbreviations are used in this manuscript:

I-PACE	Interaction of Person-Affect-Cognition-Execution
CIUT	Compensatory Internet Use Theory
FoMO	Fear of Missing Out
IAD	Internet Addiction Disorder
IGD	Internet Gaming Disorder
SMA	Social Media Addiction
ICD-11	International Classification of Diseases
MAC	Meaning and Attentional Components model
BFM	The Boredom Feedback Model
BPS	Boredom Proneness Scale
PRISMA	Preferred Reporting Items for Systematic Reviews and Meta-Analyses
PROSPERO	International Prospective Register of Systematic Reviews
NIH	National Institutes of Health
SD	Standard deviation
IAS	Internet Addiction Scale
SELSA	Social and Emotional Loneliness Scale
HADS	Hospital Anxiety and Depression Scale
MCQ-30	Metacognitions Questionnaire 30
IAT	The Internet Addiction Test
SPSRQ	The Sensitivity to Punishment and Sensitivity to Reward Questionnaire
PIU	Problematic Internet Use
ZBS	Boredom Susceptibility Scale
CPGI	The Canadian Problem Gambling Index
DQ	Five-item Dissociation Questionnaire
BIS-SF	Barratt Impulsivity Scale–Short Form
BPS-SF	Boredom Proneness Scale-Short Form
PSU	Problematic Smartphone Use
PMPU	Problematic Mobile Phone Use
PFU	Problematic Use of Facebook
PCI	Pornography Consumption Inventory
PSNSU	Problematic Social Networking Sites Use
PMSMU	Problematic Mobile Social Media Use
ADHD	Hyperactivity Disorder
HSDI	Validation of Hungarian Smartphone Deprivation Inventory
SAS	Smartphone Addiction Scale
SPAI	the Smartphone Addiction Inventory
DASS-21	the Depression Anxiety Stress Scales
PIU-Q	Problem Internet Use Questionnaire
UCLA	Loneliness Self-reporting Scale
DTS	The Distress Tolerance Scale
PDMS	Positive Drinking Expectancy Scale
s-IAT-ICD	Short Internet Addiction Test for Internet-Communication Disorder
IUES	Internet-Use Expectancies Scale
RTSQ	Ruminative thought style questionnaire
HSNS	Hypersensitive Narcissism Scale
BSSS-8	Brief Sensation Seeking Scale
RSES	Rosenberg Self-Esteem Scale
CD-RISC-10	10-item Connor–Davidson Resilience Scale
SSS	Sensation seeking scale
SAS-SV	The 10-item Smartphone Addiction Scale-Short Version
CES-D	Center for Epidemiologic Studies Depression Scale
MPAI	The Mobile Phone Addiction Index
SUF	Smartphone Use Frequency Scale
RRS	Ruminative Responses Scale
UPPS-P	The Short Impulsive Behavior Scale
MTUAS	The Anxiety/Dependence on Technology subscale of the Media and Technology Usage and Attitudes Scale
MAAS	The Mindful Attention Awareness Scale
SCS	Self-control Scale
TAS-20	Toronto Alexithymia-20 Scale
SIAS	Social Interaction Anxiousness Scale
BIS-15	Barratt Impulsiveness Scale-15
MSUQ	Metacognitions about Smartphone Use Questionnaire
SUES	Smartphone Use Expectancies Scale
BFAS	The Bergen Facebook Addiction Scale
MSBS-SF	The Multidimensional State Boredom Scale-Short Form
CSES	Core self-evaluations scale
BPS	Bedtime Procrastination Scale
MPATS	Mobile phone addiction tendency scale
PHQ-9	The Patient Health Questionnaire-9
SIAS	The Social Interaction Anxiety Scale
DIS	The Distress Intolerance Scale
PERS	The Perth Emotional Reactivity Scale-Short Form
MDTQ	Metacognitions about Desire Thinking Questionnaire
DTQ	Desire Thinking Questionnaire
PACS-SNSs	Penn Alcohol Craving Scale
BSMAS	Bergen Social Media Addiction Scale
PMSMUS	Problematic Mobile Social Media Use Scale
Y-BOCS	Yale–Brown obsessive–compulsive scale
HB	High Boredom
LB	Low Boredom
CBT	Cognitive Behavioral Therapy
ADHD	Attention Deficit/Hyperactivity Disorder

## Appendix A

**Table A1:** PRISMA 2020 checklist.

Section and Topic	Item	Checklist Item	Location Where Item Is Reported
TITLE			
Title	1	Identify the report as a systematic review	Page 1
ABSTRACT			
Abstract	2	Report an abstract addressing each item in the PRISMA 2020 for Abstracts checklist	Page 1
INTRODUCTION			
Rationale	3	Describe the rationale for the review in the context of existing knowledge	Page 2
Objectives	4	Provide an explicit statement of the objective(s) or question(s) the review addresses	Page 4
METHODS			
Eligibility criteria	5	Specify the inclusion and exclusion criteria for the review and how studies were grouped for the syntheses	Page 5
Information sources	6	Specify all databases, registers, websites, organisations, reference lists, and other sources searched or consulted to identify studies. Specify the date when each source was last searched or consulted	Page 5
Search strategy	7	Present the full search strategies for all databases, registers, and websites, including any filters and limits used	Page 5
Selection process	8	Specify the methods used to decide whether a study met the inclusion criteria of the review, including how many reviewers screened each record and each report retrieved, whether they worked independently, and, if applicable, details of automation tools used in the process	Page 5
Data collection process	9	Specify the methods used to collect data from reports, including how many reviewers collected data from each report, whether they worked independently, any processes for obtaining or confirming data from study investigators, and, if applicable, details of automation tools used in the process	Page 5
Data items	10a	List and define all outcomes for which data were sought. Specify whether all results that were compatible with each outcome domain in each study were sought (for example, for all measures, time points, analyses), and, if not, the methods used to decide which results to collect	Page 5
Data items	10b	List and define all other variables for which data were sought (such as participant and intervention characteristics, funding sources). Describe any assumptions made about any missing or unclear information	Page 5
Study risk of bias assessment	11	Specify the methods used to assess risk of bias in the included studies, including details of the tool(s) used, how many reviewers assessed each study and whether they worked independently, and, if applicable, details of automation tools used in the process	Page 6
Effect measures	12	Specify for each outcome the effect measure(s) (such as risk ratio, mean difference) used in the synthesis or presentation of results	NA
Synthesis methods	13a	Describe the processes used to decide which studies were eligible for each synthesis (such as tabulating the study intervention characteristics and comparing against the planned groups for each synthesis (item #5))	Page 5
Synthesis methods	13b	Describe any methods required to prepare the data for presentation or synthesis, such as handling of missing summary statistics or data conversions	-
Synthesis methods	13c	Describe any methods used to tabulate or visually display results of individual studies and syntheses	Page 9–15
Synthesis methods	13d	Describe any methods used to synthesise results and provide a rationale for the choice(s). If meta-analysis was performed, describe the model(s), method(s) to identify the presence and extent of statistical heterogeneity, and software package(s) used	-
Synthesis methods	13e	Describe any methods used to explore possible causes of heterogeneity among study results (such as subgroup analysis, meta-regression)	NA
Synthesis methods	13f	Describe any sensitivity analyses conducted to assess robustness of the synthesised results	-
Reporting bias assessment	14	Describe any methods used to assess risk of bias due to missing results in a synthesis (arising from reporting biases)	Page 6–8
Certainty assessment	15	Describe any methods used to assess certainty (or confidence) in the body of evidence for an outcome	Page 6–8
RESULTS			
Study selection	16a	Describe the results of the search and selection process, from the number of records identified in the search to the number of studies included in the review, ideally using a flow diagram https://www.prisma-statement.org/prisma-2020 (accessed on 21 July 2024)	Page 5–6
Study selection	16b	Cite studies that might appear to meet the inclusion criteria, but which were excluded, and explain why they were excluded	-
Study characteristics	17	Cite each included study and present its characteristics	Page 9–15
Risk of bias in studies	18	Present assessments of risk of bias for each included study	Page 6–8
Results of individual studies	19	For all outcomes, present for each study (a) summary statistics for each group (where appropriate) and (b) an effect estimate and its precision (such as confidence/credible interval), ideally using structured tables or plots	Page 9–15
Results of syntheses	20a	For each synthesis, briefly summarise the characteristics and risk of bias among contributing studies	Page 9–15
Results of syntheses	20b	Present results of all statistical syntheses conducted. If meta-analysis was done, present for each the summary estimate and its precision (such as confidence/credible interval) and measures of statistical heterogeneity. If comparing groups, describe the direction of the effect	NA
Reporting biases	21	Present results of all investigations of possible causes of heterogeneity among study results	NA
Certainty of evidence	22	Present results of all sensitivity analyses conducted to assess the robustness of the synthesised results	-
DISCUSSION			
Results in context	23	Provide a general interpretation of the results in the context of other evidence	Page 18–22
Limitations of included studies	24	Discuss any limitations of the evidence included in the review	Page 22–23
Limitations of the review methods	25	Discuss any limitations of the review processes used	Page 22–23
Implications	26	Discuss implications of the results for practice, policy, and future research	Page 23
OTHER INFORMATION			
Registration and protocol	27a	Provide registration information for the review, including register name and registration number, or state that the review was not registered	Page 5
Registration and protocol	27b	Indicate where the review protocol can be accessed, or state that a protocol was not prepared	Page 5
Registration and protocol	27c	Describe and explain any amendments to information provided at registration or in the protocol	-
Support	28	Describe sources of financial or non-financial support for the review, and the role of the funders or sponsors in the review	Page 23
Competing interests	29	Declare any competing interests of review authors	Page 23
Availability of data, code, and other materials	30	Report which of the following are publicly available and where they can be found: template data collection forms; data extracted from included studies; data used for all analyses; analytic code; any other materials used in the review	-

NA, Not Applicable. a–f: The letters following the number indicate subsections of the same guideline item, each addressing a specific but related aspect.

## References

[1] Widyanto L., Griffiths M. (2006). ‘Internet Addiction’: A Critical Review. Int. J. Ment. Health Addict..

[2] Marks I. (1990). Behavioural (Non-Chemical) Addictions. Br. J. Addict..

[3] Griffiths M. (1995). University of Plymouth Technological Addictions. Clin. Psychol. Forum.

[4] Griffiths M. (1996). Internet “Addiction”: An Issue for Clinical Psychology?. Br. Psychol. Soc..

[5] Young K.S. (1999). Internet Addiction: Evaluation and Treatment. BMJ.

[6] LaRose R., Connolly R., Lee H., Li K., Kayla K.D. (2014). Connection Overload? A Cross Cultural Study of the Consequences of Social Media Connection. Inf. Syst. Manag..

[7] Ryan T., Chester A., Reece J., Xenos S. (2014). The Uses and Abuses of Facebook: A Review of Facebook Addiction. J. Behav. Addict..

[8] Reed G.M., First M.B., Billieux J., Cloitre M., Briken P., Achab S., Brewin C.R., King D.L., Kraus S.W., Bryant R.A. (2022). Emerging Experience with Selected New Categories in the ICD-11: Complex PTSD, Prolonged Grief Disorder, Gaming Disorder, and Compulsive Sexual Behaviour Disorder. World Psychiatry.

[9] APA (2023). The American Psychiatric Association Practice Guideline for the Treatment of Patients with Eating Disorders. Am. J. Psychiatry.

[10] Elhai J.D., Gallinari E.F., Rozgonjuk D., Yang H. (2020). Depression, Anxiety and Fear of Missing out as Correlates of Social, Non-Social and Problematic Smartphone Use. Addict. Behav..

[11] Chotpitayasunondh V., Douglas K.M. (2016). How “Phubbing” Becomes the Norm: The Antecedents and Consequences of Snubbing via Smartphone. Comput. Hum. Behav..

[12] Gentile D.A., Choo H., Liau A., Sim T., Li D., Fung D., Khoo A. (2011). Pathological Video Game Use Among Youths: A Two-Year Longitudinal Study. Pediatrics.

[13] Savci M., Aysan F. (2016). Relationship between Impulsivity, Social Media Usage and Loneliness. Educ. Process Int. J..

[14] Kiss H., Fitzpatrick K.M., Piko B.F. (2020). The Digital Divide: Risk and Protective Factors and the Differences in Problematic Use of Digital Devices among Hungarian Youth. Child. Youth Serv. Rev..

[15] Donati M.A., Beccari C., Primi C. (2022). Boredom and Problematic Facebook Use in Adolescents: What Is the Relationship Considering Trait or State Boredom?. Addict. Behav..

[16] Larche C.J., Dixon M.J. (2021). Winning Isn’t Everything: The Impact of Optimally Challenging Smartphone Games on Flow, Game Preference and Individuals Gaming to Escape Aversive Bored States. Comput. Hum. Behav..

[17] Eastwood J.D., Frischen A., Fenske M.J., Smilek D. (2012). The Unengaged Mind: Defining Boredom in Terms of Attention. Perspect. Psychol. Sci..

[18] Neu J. (2000). Boring From Within: Endogenous versus Reactive Boredom. A Tear Is an Intellectual Thing.

[19] Elpidorou A. (2018). The Bored Mind Is a Guiding Mind: Toward a Regulatory Theory of Boredom. Phenomenol. Cogn. Sci..

[20] Mercer-Lynn K.B., Bar R.J., Eastwood J.D. (2014). Causes of Boredom: The Person, the Situation, or Both?. Personal. Individ. Differ..

[21] Farmer R., Sundberg N.D. (1986). Boredom Proneness-The Development and Correlates of a New Scale. J. Pers. Assess..

[22] Gorelik D., Eastwood J.D. (2024). Trait Boredom as a Lack of Agency: A Theoretical Model and a New Assessment Tool. Assessment.

[23] Danckert J., Eastwood J.D. (2020). Out of My Skull: The Psychology of Boredom.

[24] Fahlman S.A., Mercer-Lynn K.B., Flora D.B., Eastwood J.D. (2013). Development and Validation of the Multidimensional State Boredom Scale. Assessment.

[25] Goldberg Y.K., Eastwood J.D., LaGuardia J., Danckert J. (2011). Boredom: An Emotional Experience Distinct from Apathy, Anhedonia, or Depression. J. Soc. Clin. Psychol..

[26] Li X., Feng X., Xiao W., Zhou H. (2021). Loneliness and Mobile Phone Addiction Among Chinese College Students: The Mediating Roles of Boredom Proneness and Self-Control. Psychol. Res. Behav. Manag..

[27] Mercer-Lynn K.B., Flora D.B., Fahlman S.A., Eastwood J.D. (2013). The Measurement of Boredom: Differences Between Existing Self-Report Scales. Assessment.

[28] Yang X.-J., Liu Q.-Q., Lian S.-L., Zhou Z.-K. (2020). Are Bored Minds More Likely to Be Addicted? The Relationship between Boredom Proneness and Problematic Mobile Phone Use. Addict. Behav..

[29] Xiao W., Zhou H., Li X., Lin X. (2021). Why Are Individuals with Alexithymia Symptoms More Likely to Have Mobile Phone Addiction? The Multiple Mediating Roles of Social Interaction Anxiousness and Boredom Proneness. Psychol. Res. Behav. Manag..

[30] Westgate E.C., Wilson T.D. (2018). Boring Thoughts and Bored Minds: The MAC Model of Boredom and Cognitive Engagement. Psychol. Rev..

[31] Westgate E.C. (2020). Why Boredom Is Interesting. Curr. Dir. Psychol. Sci..

[32] Poels K., Rudnicki K., Vandebosch H. (2022). The Media Psychology of Boredom and Mobile Media Use. J. Media Psychol..

[33] Tam K.Y.Y., van Tilburg W.A.P., Chan C.S. (2021). What Is Boredom Proneness? A Comparison of Three Characterizations. J. Pers..

[34] Brand M., Wegmann E., Stark R., Müller A., Wölfling K., Robbins T.W., Potenza M.N. (2019). The Interaction of Person-Affect-Cognition-Execution (I-PACE) Model for Addictive Behaviors: Update, Generalization to Addictive Behaviors beyond Internet-Use Disorders, and Specification of the Process Character of Addictive Behaviors. Neurosci. Biobehav. Rev..

[35] Orsolini L., Longo G., Volpe U. (2023). The Mediatory Role of the Boredom and Loneliness Dimensions in the Development of Problematic Internet Use. Int. J. Environ. Res. Public Health.

[36] Zhang N., Li J. (2022). Effect and Mechanisms of State Boredom on Consumers’ Livestreaming Addiction. Front. Psychol..

[37] Freund V.A., Schulenberg J.E., Maslowsky J. (2021). Boredom by Sensation-Seeking Interactions During Adolescence: Associations with Substance Use, Externalizing Behavior, and Internalizing Symptoms in a US National Sample. Prev. Sci..

[38] Biolcati R., Mancini G., Trombini E. (2018). Proneness to Boredom and Risk Behaviors During Adolescents’ Free Time. Psychol. Rep..

[39] Camerini A.-L., Morlino S., Marciano L. (2023). Boredom and Digital Media Use: A Systematic Review and Meta-Analysis. Comput. Hum. Behav. Rep..

[40] Struk A.A., Carriere J.S.A., Cheyne J.A., Danckert J. (2017). A Short Boredom Proneness Scale: Development and Psychometric Properties. Assessment.

[41] Drody A.C., Ralph B.C.W., Danckert J., Smilek D. (2022). Boredom and Media Multitasking. Front. Psychol..

[42] Page M.J., McKenzie J.E., Bossuyt P.M., Boutron I., Hoffmann T.C., Mulrow C.D., Shamseer L., Tetzlaff J.M., Akl E.A., Brennan S.E. (2021). The PRISMA 2020 Statement: An Updated Guideline for Reporting Systematic Reviews. BMJ.

[43] Panic N., Leoncini E., de Belvis G., Ricciardi W., Boccia S. (2013). Evaluation of the Endorsement of the Preferred Reporting Items for Systematic Reviews and Meta-Analysis (PRISMA) Statement on the Quality of Published Systematic Review and Meta-Analyses. PLoS ONE.

[44] Brodeur A., Clark A.E., Fleche S., Powdthavee N. (2021). COVID-19, Lockdowns and Well-Being: Evidence from Google Trends. J. Public Econ..

[45] Hu J., Zhao C., Yu T. (2025). The Relationship Between Boredom and Smartphone Addiction Before and After the Outbreak of the COVID-19 Pandemic: A Systematic Review and Meta-Analysis. Psychol. Rep..

[46] Elhai J.D., Levine J.C., Alghraibeh A.M., Alafnan A.A., Aldraiweesh A.A., Hall B.J. (2018). Fear of Missing out: Testing Relationships with Negative Affectivity, Online Social Engagement, and Problematic Smartphone Use. Comput. Hum. Behav..

[47] Elhai J.D., Vasquez J.K., Lustgarten S.D., Levine J.C., Hall B.J. (2018). Proneness to Boredom Mediates Relationships Between Problematic Smartphone Use With Depression and Anxiety Severity. Soc. Sci. Comput. Rev..

[48] Skues J., Williams B., Oldmeadow J., Wise L. (2016). The Effects of Boredom, Loneliness, and Distress Tolerance on Problem Internet Use Among University Students. Int. J. Ment. Health Addict..

[49] Hopley A.A.B., Nicki R.M. (2010). Predictive Factors of Excessive Online Poker Playing. Cyberpsychol. Behav. Soc. Netw..

[50] Regan T., Harris B., Loon M.V., Nanavaty N., Schueler J., Engler S., Fields S.A. (2020). Does Mindfulness Reduce the Effects of Risk Factors for Problematic Smartphone Use? Comparing Frequency of Use versus Self-Reported Addiction. Addict. Behav..

[51] Wegmann E., Ostendorf S., Brand M. (2018). Is It Beneficial to Use Internet-Communication for Escaping from Boredom? Boredom Proneness Interacts with Cue-Induced Craving and Avoidance Expectancies in Explaining Symptoms of Internet-Communication Disorder. PLoS ONE.

[52] Holte A.J., Aukerman K., Padgett R., Kenna M. (2024). “Let Me Check My Phone Just One More Time”: Understanding the Relationship of Obsessive-Compulsive Disorder Severity and Problematic Smartphone Use. Curr. Psychol..

[53] Mercer K.B., Eastwood J.D. (2010). Is Boredom Associated with Problem Gambling Behaviour? It Depends on What You Mean by ‘Boredom.’. Int. Gambl. Stud..

[54] Spada M.M., Langston B., Nikčević A.V., Moneta G.B. (2008). The Role of Metacognitions in Problematic Internet Use. Comput. Hum. Behav..

[55] Bai J., Mo K., Peng Y., Hao W., Qu Y., Lei X., Yang Y. (2021). The Relationship Between the Use of Mobile Social Media and Subjective Well-Being: The Mediating Effect of Boredom Proneness. Front. Psychol..

[56] Casale S., Fioravanti G., Spada M.M. (2021). Modelling the Contribution of Metacognitions and Expectancies to Problematic Smartphone Use. J. Behav. Addict..

[57] Pi L., Wang Y., Zou L., Mo X., and L.G. (2024). An Analysis of the Latent Class and Influencing Factors of Problematic Mobile Social Media Usage Among Chinese College Students. Psychol. Res. Behav. Manag..

[58] Bocci Benucci S., Tonini B., Roffo G., Casale S., Fioravanti G. (2024). The Application of the Metacognitive Model of Desire Thinking and Craving in Problematic Social Networking Sites Use. Psychiatr. Q..

[59] Moynihan A.B., Igou E.R., van Tilburg W.A.P. (2022). Pornography Consumption as Existential Escape from Boredom. Personal. Individ. Differ..

[60] Wang Y., Yang H., Montag C., Elhai J.D. (2022). Boredom Proneness and Rumination Mediate Relationships between Depression and Anxiety with Problematic Smartphone Use Severity. Curr. Psychol..

[61] Ksinan A.J., Mališ J., Vazsonyi A.T. (2021). Swiping Away the Moments That Make up a Dull Day: Narcissism, Boredom, and Compulsive Smartphone Use. Curr. Psychol..

[62] Wang Z., Yang X., Zhang X. (2020). Relationships among Boredom Proneness, Sensation Seeking and Smartphone Addiction among Chinese College Students: Mediating Roles of Pastime, Flow Experience and Self-Regulation. Technol. Soc..

[63] Wolniewicz C.A., Rozgonjuk D., Elhai J.D. (2020). Boredom Proneness and Fear of Missing out Mediate Relations between Depression and Anxiety with Problematic Smartphone Use. Hum. Behav. Emerg. Technol..

[64] Nichols L., Nicki R. (2004). Development of a Psychometrically Sound Internet Addiction Scale: A Preliminary Step. Psychol. Addict. Behav. J. Soc. Psychol. Addict. Behav..

[65] Yao N., Chen J., Huang S., Montag C., Elhai J.D. (2023). Depression and Social Anxiety in Relation to Problematic TikTok Use Severity: The Mediating Role of Boredom Proneness and Distress Intolerance. Comput. Hum. Behav..

[66] Zhu Y., Liu J., Wang Q., Huang J., Li X., Liu J. (2023). Examining the Association Between Boredom Proneness and Bedtime Procrastination Among Chinese College Students: A Sequential Mediation Model with Mobile Phone Addiction and Negative Emotions. Psychol. Res. Behav. Manag..

[67] Li X., Zhou H., Xiao W. (2022). Boredom Proneness and Core Self-Evaluation as Mediators between Loneliness and Mobile Phone Addiction among Chinese College Students. Psychol. Sch..

[68] DiTommaso E., Spinner B. (1993). The Development and Initial Validation of the Social and Emotional Loneliness Scale for Adults (SELSA). Personal. Individ. Differ..

[69] Zigmond A.S., Snaith R.P. (1983). The Hospital Anxiety and Depression Scale. Acta Psychiatr. Scand..

[70] Wells A., Cartwright-Hatton S. (2004). A Short Form of the Metacognitions Questionnaire: Properties of the MCQ-30. Behav. Res. Ther..

[71] Zuckerman M. (1979). Sensation Seeking: Beyond the Optimal Level of Arousal.

[72] Torrubia R., Ávila C., Moltó J., Caseras X. (2001). The Sensitivity to Punishment and Sensitivity to Reward Questionnaire (SPSRQ) as a Measure of Gray’s Anxiety and Impulsivity Dimensions. Personal. Individ. Differ..

[73] Ferris J., Wynne H., Ladouceur R., Stinchfield R., Turner N. (2001). The Canadian Problem Gambling Index: Final Report.

[74] Diskin K.M., Hodgins D.C. (1999). Narrowing of Attention and Dissociation in Pathological Video Lottery Gamblers. J. Gambl. Stud..

[75] Spinella M. (2007). Normative data and a short form of the barratt impulsiveness scale. Int. J. Neurosci..

[76] Lovibond P.F., Lovibond S.H. (1995). The Structure of Negative Emotional States: Comparison of the Depression Anxiety Stress Scales (DASS) with the Beck Depression and Anxiety Inventories. Behav. Res. Ther..

[77] Demetrovics Z., Szeredi B., Rózsa S. (2008). The Three-Factor Model of Internet Addiction: The Development of the Problematic Internet Use Questionnaire. Behav. Res. Methods.

[78] Russell D.W. (1996). UCLA Loneliness Scale (Version 3): Reliability, Validity, and Factor Structure. J. Pers. Assess..

[79] Simons J.S., Gaher R.M. (2005). The Distress Tolerance Scale: Development and Validation of a Self-Report Measure. Motiv. Emot..

[80] D’Alessio M., Baiocco R., Laghi F. (2006). The Problem of Binge Drinking among Italian University Students: A Preliminary Investigation. Addict. Behav..

[81] Wegmann E., Stodt B., Brand M. (2015). Addictive Use of Social Networking Sites Can Be Explained by the Interaction of Internet Use Expectancies, Internet Literacy, and Psychopathological Symptoms. J. Behav. Addict..

[82] Wegmann E., Oberst U., Stodt B., Brand M. (2017). Online-Specific Fear of Missing out and Internet-Use Expectancies Contribute to Symptoms of Internet-Communication Disorder. Addict. Behav. Rep..

[83] Love A., James D., Willner P. (1998). A Comparison of Two Alcohol Craving Questionnaires. Addiction.

[84] Brand M., Laier C., Young K.S. (2014). Internet Addiction: Coping Styles, Expectancies, and Treatment Implications. Front. Psychol..

[85] Elhai J.D., Levine J.C., Dvorak R.D., Hall B.J. (2016). Fear of Missing out, Need for Touch, Anxiety and Depression Are Related to Problematic Smartphone Use. Comput. Hum. Behav..

[86] Kwon M., Lee J.-Y., Won W.-Y., Park J.-W., Min J.-A., Hahn C., Gu X., Choi J.-H., Kim D.-J. (2013). Development and Validation of a Smartphone Addiction Scale (SAS). PLoS ONE.

[87] Przybylski A.K., Murayama K., DeHaan C.R., Gladwell V. (2013). Motivational, Emotional, and Behavioral Correlates of Fear of Missing Out. Comput. Hum. Behav..

[88] van Deursen A.J.A.M., Bolle C.L., Hegner S.M., Kommers P.A.M. (2015). Modeling Habitual and Addictive Smartphone Behavior: The Role of Smartphone Usage Types, Emotional Intelligence, Social Stress, Self-Regulation, Age, and Gender. Comput. Hum. Behav..

[89] Brinker J.K., Campisi M., Gibbs L., Izzard R. (2013). Rumination, Mood and Cognitive Performance. Psychology.

[90] Ames D.R., Rose P., Anderson C.P. (2006). The NPI-16 as a Short Measure of Narcissism. J. Res. Personal..

[91] Hendin H.M., Cheek J.M. (1997). Assessing Hypersensitive Narcissism: A Reexamination of Murray’s Narcism Scale. J. Res. Personal..

[92] Lee Y.-K., Chang C.-T., Lin Y., Cheng Z.-H. (2014). The Dark Side of Smartphone Usage: Psychological Traits, Compulsive Behavior and Technostress. Comput. Hum. Behav..

[93] Lin Y.-H., Chang L.-R., Lee Y.-H., Tseng H.-W., Kuo T.B.J., Chen S.-H. (2014). Development and Validation of the Smartphone Addiction Inventory (SPAI). PLoS ONE.

[94] Csibi S., Demetrovics Z., Szabo A. (2017). Validation of Hungarian Smartphone Deprivation Inventory (HSDI) with School Children. Psychiatr. Hung. Magy. Pszichiatriai Tars. Tudomanyos Folyoirata.

[95] Hoyle R.H., Stephenson M.T., Palmgreen P., Lorch E.P., Donohew R.L. (2002). Reliability and Validity of a Brief Measure of Sensation Seeking. Personal. Individ. Differ..

[96] Mayer K., Lukács A., Pauler G. (2012). Hungarian Adaptation of the 8-Item Sensation Seeking Scale (BSSS-8). Mentálhig. Pszichoszomatika.

[97] Rosenberg M. (2015). Society and the Adolescent Self-Image.

[98] Luszczynska A., Diehl M., Gutiérrez-Doña B., Kuusinen P., Schwarzer R. (2004). Measuring One Component of Dispositional Self-Regulation: Attention Control in Goal Pursuit. Personal. Individ. Differ..

[99] Magyaródi T., Nagy H., Soltész P., Mózes T., Oláh A. (2013). Psychometric Properties of a Newly Established Flow State Questionnaire. J. Happiness Well-Being.

[100] Connor K.M., Davidson J.R. (2003). Development of a New Resilience Scale: The Connor-Davidson Resilience Scale (CD-RISC). Depress. Anxiety.

[101] Campbell-Sills L., Stein M.B. (2007). Psychometric Analysis and Refinement of the Connor–Davidson Resilience Scale (CD-RISC): Validation of a 10-item Measure of Resilience. J. Trauma. Stress.

[102] Róbert J., Dóra V., Rita H., László N., Krisztina C., Csilla K.E. (2015). A connor-davidson reziliencia kérdőív 10 itemes változatának jellemzői. Alkalm. Pszichológia.

[103] Wang P., Lei L., Wang X., Nie J., Chu X., Jin S. (2018). The Exacerbating Role of Perceived Social Support and the “Buffering” Role of Depression in the Relation between Sensation Seeking and Adolescent Smartphone Addiction. Personal. Individ. Differ..

[104] Chen B., Liu F., Ding S., Ying X., Wang L., Wen Y. (2017). Gender Differences in Factors Associated with Smartphone Addiction: A Cross-Sectional Study among Medical College Students. BMC Psychiatry.

[105] Liu H., Chu H., Huang Q., Chen X. (2016). Enhancing the Flow Experience of Consumers in China through Interpersonal Interaction in Social Commerce. Comput. Hum. Behav..

[106] Gökçearslan Ş., Mumcu F.K., Haşlaman T., Çevik Y.D. (2016). Modelling Smartphone Addiction: The Role of Smartphone Usage, Self-Regulation, General Self-Efficacy and Cyberloafing in University Students. Comput. Hum. Behav..

[107] Radloff L.S. (1977). The CES-D Scale: A Self-Report Depression Scale for Research in the General Population. Appl. Psychol. Meas..

[108] Leung L. (2008). Linking psychological attributes to addiction and improper use of the mobile phone among adolescents in Hong Kong. J. Child. Media.

[109] Carriere J.S., Seli P., Smilek D. (2013). Wandering in Both Mind and Body: Individual Differences in Mind Wandering and Inattention Predict Fidgeting. Can. J. Exp. Psychol. Can. Psychol. Expérimentale.

[110] Nolen-Hoeksema S., Wisco B.E., Lyubomirsky S. (2008). Rethinking Rumination. Perspect. Psychol. Sci..

[111] Cyders M.A., Littlefield A.K., Coffey S., Karyadi K.A. (2014). Examination of a Short English Version of the UPPS-P Impulsive Behavior Scale. Addict. Behav..

[112] Rosen L.D., Whaling K., Carrier L.M., Cheever N.A., Rokkum J. (2013). The Media and Technology Usage and Attitudes Scale: An Empirical Investigation. Comput. Hum. Behav..

[113] Brown K.W., Ryan R.M. (2003). The Benefits of Being Present: Mindfulness and Its Role in Psychological Well-Being. J. Pers. Soc. Psychol..

[114] Tangney J.P., Baumeister R.F., Boone A.L. (2004). High Self-Control Predicts Good Adjustment, Less Pathology, Better Grades, and Interpersonal Success. J. Pers..

[115] Bagby R.M., Parker J.D.A., Taylor G.J. (1994). The Twenty-Item Toronto Alexithymia Scale-I. Item Selection and Cross-Validation of the Factor Structure. J. Psychosom. Res..

[116] Leary M.R., Kowalski R.M. (1993). The Interaction Anxiousness Scale: Construct and Criterion-Related Validity. J. Pers. Assess..

[117] Liang C., Sun J. (2022). A Study of the Happiness of Chinese University Students and Its Influencing Factors-A Case Study of Beijing Universities. Sustainability.

[118] Bottesi G., Ghisi M., Altoè G., Conforti E., Melli G., Sica C. (2015). The Italian Version of the Depression Anxiety Stress Scales-21: Factor Structure and Psychometric Properties on Community and Clinical Samples. Compr. Psychiatry.

[119] Patton J.H., Stanford M.S., Barratt E.S. (1995). Factor Structure of the Barratt Impulsiveness Scale. J. Clin. Psychol..

[120] Casale S., Caponi L., Fioravanti G. (2020). Metacognitions about Problematic Smartphone Use: Development of a Self-Report Measure. Addict. Behav..

[121] De Pasquale C., Sciacca F., Hichy Z. (2017). Italian Validation of Smartphone Addiction Scale Short Version for Adolescents and Young Adults (SAS-SV). Psychology.

[122] Andreassen C.S., Torsheim T., Brunborg G.S., Pallesen S. (2012). Development of a Facebook Addiction Scale. Psychol. Rep..

[123] Soraci P., Ferrari A., Barberis N., Luvarà G., Urso A., Del Fante E., Griffiths M.D. (2023). Psychometric Analysis and Validation of the Italian Bergen Facebook Addiction Scale. Int. J. Ment. Health Addict..

[124] Hunter J.A., Dyer K.J., Cribbie R.A., Eastwood J.D. (2016). Exploring the Utility of the Multidimensional State Boredom Scale. Eur. J. Psychol. Assess..

[125] Judge T.A., Erez A., Bono J.E., Thoresen C.J. (2003). The core self-evaluations scale: Development of a measure. Pers. Psychol..

[126] Steger M.F., Frazier P., Oishi S., Kaler M. (2006). The Meaning in Life Questionnaire: Assessing the Presence of and Search for Meaning in Life. J. Couns. Psychol..

[127] Reid R.C., Li D.S., Gilliland R., Stein J.A., Fong T. (2011). Reliability, Validity, and Psychometric Development of the Pornography Consumption Inventory in a Sample of Hypersexual Men. J. Sex Marital Ther..

[128] Li Y.-Y., Huang Y.-T., Dou K. (2021). Validation and Psychometric Properties of the Chinese Version of the Fear of Missing Out Scale. Int. J. Environ. Res. Public Health.

[129] Ma X., Meng D., Zhu L., Xu H., Guo J., Yang L., Yu L., Fu Y., Mu L. (2020). Bedtime Procrastination Predicts the Prevalence and Severity of Poor Sleep Quality of Chinese Undergraduate Students. J. Am. Coll. Health.

[130] Xiong J., Zhou Z.-K., Chen W., You Z.-Q., Zhai Z.-Y. (2012). Mobile Phone Addiction Tendency Scale.

[131] Gong X., Xie X., Xu R., Luo Y. (2010). Psychometric Properties of the Chinese Versions of DASS-21 in Chinese College Students. Chin. J. Clin. Psychol..

[132] Kroenke K., Spitzer R.L., Williams J.B.W. (2001). The PHQ-9. J. Gen. Intern. Med..

[133] Bian C., Li C., Duan Q., Wu H. (2011). Reliability and Validity of Patient Health Questionnaire: Depressive Syndrome Module for Outpatients. Sci. Res. Essays.

[134] Mattick R.P., Clarke J.C. (1998). Development and validation of measures of social phobia scrutiny fear and social interaction anxiety. Behav. Res. Ther..

[135] Ye D., Qian M., Liu X., Chen X. (2007). Revision of Social Interaction Anxiety Scale and Social Phobia Scale. Chin. J. Clin. Psychol..

[136] McHugh R.K., Otto M.W. (2012). Refining the Measurement of Distress Intolerance. Behav. Ther..

[137] Preece D., Becerra R., Campitelli G. (2019). Assessing Emotional Reactivity: Psychometric Properties of the Perth Emotional Reactivity Scale and the Development of a Short Form. J. Pers. Assess..

[138] Caselli G., Spada M.M. (2015). Desire Thinking: What Is It and What Drives It?. Addict. Behav..

[139] Caselli G., Spada M.M. (2011). The Desire Thinking Questionnaire: Development and Psychometric Properties. Addict. Behav..

[140] Hormes J.M., Kearns B., Timko C.A. (2014). Craving Facebook? Behavioral Addiction to Online Social Networking and Its Association with Emotion Regulation Deficits. Addiction.

[141] Brandtner A., Wegmann E. (2023). The Fear in Desire: Linking Desire Thinking and Fear of Missing out in the Social Media Context. BMC Psychol..

[142] Cui X.X., Sun X.J., Niu G.F. (2016). The Effect of Self-Presentation in Online Social Networking Sites on Adolescents’ Friendship Quality: The Mediating Role of Positive Feedback. Psychol. Dev. Educ..

[143] Goodman W.K., Price L.H., Rasmussen S.A., Mazure C., Fleischmann R.L., Hill C.L., Heninger G.R., Charney D.S. (1989). The Yale-Brown Obsessive Compulsive Scale: I. Development, Use, and Reliability. Arch. Gen. Psychiatry.

[144] Carleton R.N., Norton M.A.P.J., Asmundson G.J.G. (2007). Fearing the Unknown: A Short Version of the Intolerance of Uncertainty Scale. J. Anxiety Disord..

[145] Kardefelt-Winther D. (2014). A Conceptual and Methodological Critique of Internet Addiction Research: Towards a Model of Compensatory Internet Use. Comput. Hum. Behav..

[146] Wölfling K., Beutel M.E., Dreier M., Müller K.W. (2014). Treatment Outcomes in Patients with Internet Addiction: A Clinical Pilot Study on the Effects of a Cognitive-Behavioral Therapy Program. BioMed. Res. Int..

[147] Han D.H., Kim S.M., Lee Y.S., Renshaw P.F. (2012). The Effect of Family Therapy on the Changes in the Severity of On-Line Game Play and Brain Activity in Adolescents with on-Line Game Addiction. Psychiatry Res. Neuroimaging.

[148] Zhang J.-T., Yao Y.-W., Potenza M.N., Xia C.-C., Lan J., Liu L., Wang L.-J., Liu B., Ma S.-S., Fang X.-Y. (2016). Altered Resting-State Neural Activity and Changes Following a Craving Behavioral Intervention for Internet Gaming Disorder. Sci. Rep..

[149] Han D.H., Lee Y.S., Na C., Ahn J.Y., Chung U.S., Daniels M.A., Haws C.A., Renshaw P.F. (2009). The Effect of Methylphenidate on Internet Video Game Play in Children with Attention-Deficit/Hyperactivity Disorder. Compr. Psychiatry.

[150] Han D.H., Renshaw P.F. (2012). Bupropion in the Treatment of Problematic Online Game Play in Patients with Major Depressive Disorder. J. Psychopharmacol..

[151] Demirtepe-Saygili D. (2022). Stress, Coping, and Social Media Use. Research Anthology on Combating Cyber-Aggression and Online Negativity.

[152] Hurley L.N. (2018). The Relationship Between Resilience, Coping, and Social Media. Master’s Thesis.

[153] Sun X., Li B.J., Zhang H., Zhang G. (2023). Social Media Use for Coping with Stress and Psychological Adjustment: A Transactional Model of Stress and Coping Perspective. Front. Psychol..

[154] Tarafdar M., Maier C., Laumer S., Weitzel T. (2020). Explaining the Link between Technostress and Technology Addiction for Social Networking Sites: A Study of Distraction as a Coping Behavior. Inf. Syst. J..

[155] Wolfers L.N., Utz S. (2022). Social Media Use, Stress, and Coping. Curr. Opin. Psychol..

[156] Sriwilai K., Charoensukmongkol P. (2016). Face It, Don’t Facebook It: Impacts of Social Media Addiction on Mindfulness, Coping Strategies and the Consequence on Emotional Exhaustion. Stress Health.

