# Supplementary material for: Connected by Boredom: A Systematic Review of the Role of Trait Boredom in Problematic Technology Use
Source: Brain Sci. 2025 Jul 25;15(8):794. doi: 10.3390/brainsci15080794 (PMC12384929; doi:10.3390/brainsci15080794)
Supplement: Supplementary file 1 [file brainsci-15-00794-s001.zip › brainsci-3739555-Table S1.pdf]

**Table S1.** GRADE System

| Study | Recommendation Grade | Evidence Level |
|---|---|---|
| [29] | 3 | C |
| [67] | 4 | D |
| [63] | 3 | C |
| [60] | 3 | C |
| [50] | 4 | D |
| [66] | 3 | C |
| [46] | 4 | D |
| [52] | 3 | D |
| [27] | 4 | C |
| [47] | 3 | C |
| [62] | 4 | D |
| [61] | 3 | C |
| [15] | 3 | B |
| [30] | 3 | C |
| [57] | 3 | C |
| [16] | 4 | D |
| [65] | 3 | C |
| [58] | 3 | A |
| [55] | 3 | C |
| [64] | 4 | D |
| [51] | 4 | C |
| [39] | 4 | C |
| [48] | 3 | C |
| [54] | 4 | C |
| [53] | 4 | D |
| [59] | 3 | C |
| [49] | 3 | D |
| [56] | 3 | C |

***Evidence Level:*** *A = High; B = Moderate; C = Low; D = Very Low*

***Recommendation Grade:*** *1 = Strong; 2 = Moderate; 3 = Weak; 4 = Very Weak*
