# Supplementary material for: Connected by Boredom: A Systematic Review of the Role of Trait Boredom in Problematic Technology Use
Source: Brain Sci. 2025 Jul 25;15(8):794. doi: 10.3390/brainsci15080794 (PMC12384929; doi:10.3390/brainsci15080794)

Figure S1. Graphical representation illustrating the mediating and moderating role of trait boredom

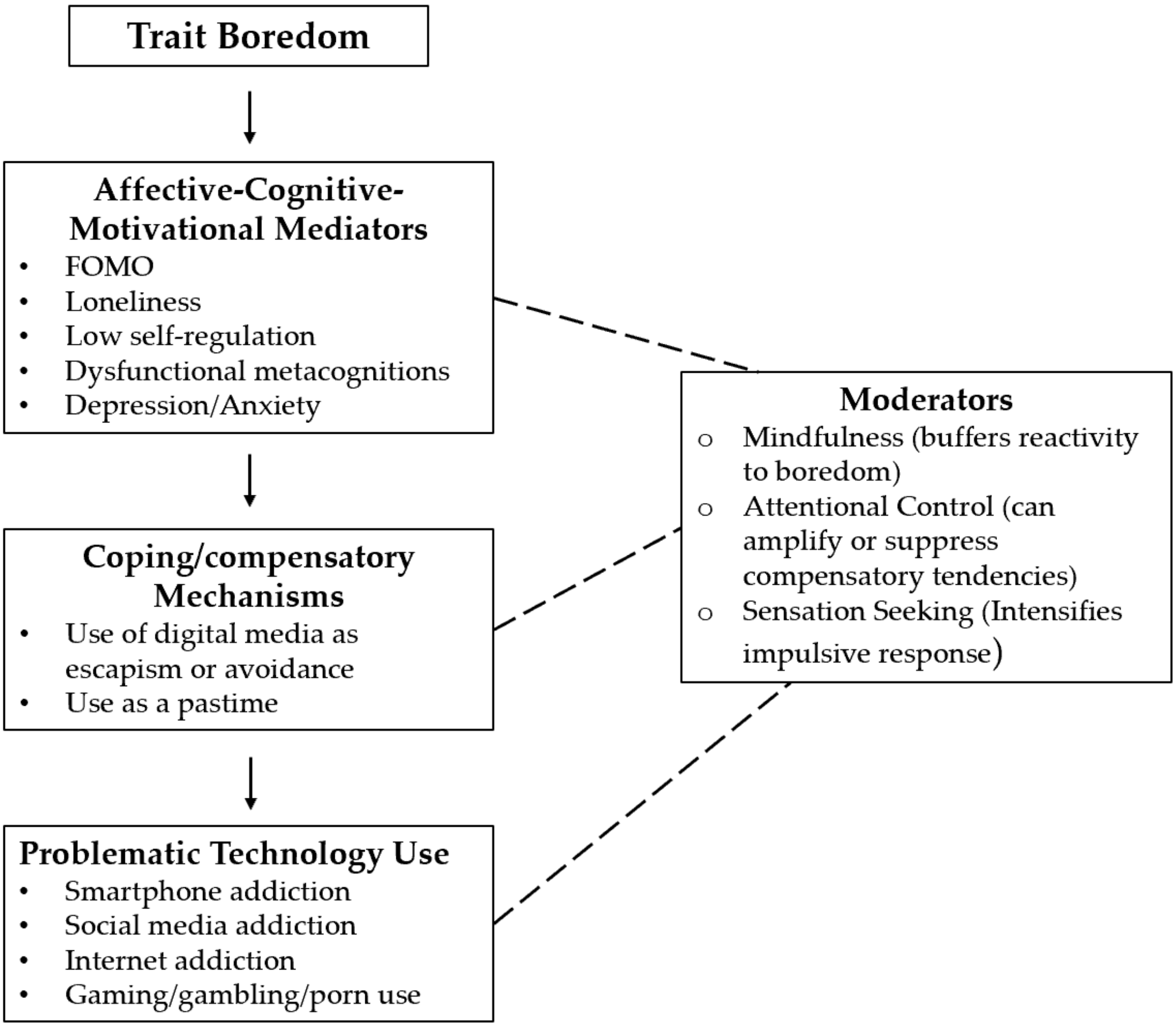

Supplement: Supplementary file 1 [file brainsci-15-00794-s001.zip › brainsci-3739555-Figure S1.pdf]
